# BioMiCo: a supervised Bayesian model for inference of microbial community structure

**DOI:** 10.1186/s40168-015-0073-x

**Published:** 2015-03-10

**Authors:** Mahdi Shafiei, Katherine A Dunn, Eva Boon, Shelley M MacDonald, David A Walsh, Hong Gu, Joseph P Bielawski

**Affiliations:** Department of Mathematics and Statistics, Dalhousie University, Halifax, NS Canada; Department of Biology, Dalhousie University, Halifax, NS Canada; Department of Biology, Concordia University, Montreal, Quebec Canada

**Keywords:** Microbial community structure, Bayesian model, Admixture model, Hierarchical mixed-membership model, Supervised learning, OTU abundance data, Microbiome, Human, Temperate coastal ocean

## Abstract

**Background:**

Microbiome samples often represent mixtures of communities, where each community is composed of overlapping assemblages of species. Such mixtures are complex, the number of species is huge and abundance information for many species is often sparse. Classical methods have a limited value for identifying complex features within such data.

**Results:**

Here, we describe a novel hierarchical model for Bayesian inference of microbial communities (BioMiCo). The model takes abundance data derived from environmental DNA, and models the composition of each sample by a two-level hierarchy of mixture distributions constrained by Dirichlet priors. BioMiCo is supervised, using known features for samples and appropriate prior constraints to overcome the challenges posed by many variables, sparse data, and large numbers of rare species. The model is trained on a portion of the data, where it learns how assemblages of species are mixed to form communities and how assemblages are related to the known features of each sample. Training yields a model that can predict the features of new samples. We used BioMiCo to build models for three serially sampled datasets and tested their predictive accuracy across different time points. The first model was trained to predict both body site (hand, mouth, and gut) and individual human host. It was able to reliably distinguish these features across different time points. The second was trained on vaginal microbiomes to predict both the Nugent score and individual human host. We found that women having normal and elevated Nugent scores had distinct microbiome structures that persisted over time, with additional structure within women having elevated scores. The third was trained for the purpose of assessing seasonal transitions in a coastal bacterial community. Application of this model to a high-resolution time series permitted us to track the rate and time of community succession and accurately predict known ecosystem-level events.

**Conclusion:**

BioMiCo provides a framework for learning the structure of microbial communities and for making predictions based on microbial assemblages. By training on carefully chosen features (abiotic or biotic), BioMiCo can be used to understand and predict transitions between complex communities composed of hundreds of microbial species.

**Electronic supplementary material:**

The online version of this article (doi:10.1186/s40168-015-0073-x) contains supplementary material, which is available to authorized users.

## Background

Microbial communities are highly complex assemblages of individual organisms. Hundreds, and sometimes thousands, of species can contribute to a community (for example, [1,2]), with the individuals belonging to a species having a wide range of interactions with other individuals in that community [3]. Moreover, the species within a community can be structured hierarchically into assemblages. For example, among all species that contribute to the human gut microbiome, only a subset have stable co-occurrence relationships, with only a further subset seeming to have stability over decades [4]. Although single species, or strains, have been successfully targeted as causative agents of disease (for example, [5]), there is growing interest to learn how the broader composition of a community might be related to some feature of concern (for example, an ecosystem process [6], the severity of a disease state [7], the impact of a dietary intervention [8], or source tracking [9]). In settings where community-level function is thought to be important, the co-occurrence relationships among lineages may be more informative than the simple presence or absence of one, or a few, indicator species [10]. Although sampling community composition via high-throughput amplicon sequencing, or via shotgun metagenomics, is no longer methodologically challenging, the data still pose a significant analytical challenge. Associations within such data are complex, the number of variables is huge, and species abundance information is sparse for many species (or strains) over many samples. In this setting, classic testing procedures have limited ability to identify complex features within the data [11,12].

Statistical model-based supervised learning is ideally suited to the challenges posed by microbial community data. This family of techniques is designed to learn the variables of a model most suited to discriminating among user-defined features of interest. Especially relevant to microbial community data is their capacity for (*i*) learning from very high-dimensional data and (*ii*) quantifying the accuracy of a model at predicting the features of concern in future datasets [11]. Despite these advantages, the existing techniques have only recently been applied to microbial community data, and very little development has been done purposely for microbial community data (but see [9,13]). Readers are referred to Knights *et al*. [12] for a thorough review of how the standard techniques can be applied to microbial community data.

Here, we focus on the task of building and evaluating a predictive model for microbiome composition data. Taking clinical microbiomics as an example, a typical research goal might be to learn how microbiome structure relates to, say, the probability of disease, and then to use that information to monitor and make predictions about an individual’s chance of disease according to his/her own microbiome samples. As a community structure is characterized by the contribution of species to assemblages, and assemblages to communities, we focus on modeling the relative abundance of both assemblages and species. Hereafter, species are defined operationally according to a sequence similarity threshold (typically 97% for 16S rRNA sequences) and are referred to as operational taxonomic units (OTUs) rather than species. Modeling compositional data within a supervised framework is not new (for example, [14]), but only two approaches have been developed purposely for modeling relative abundances of OTUs [9,13]. Both Knights *et al*. [9] and Holmes *et al*. [13] model OTU abundances by applying a Dirichlet prior to the parameters of the multinomial distribution. Knights *et al*. [9] developed their model for predicting how microbial contaminants might be mixed within a given sample (that is, source tracking). Their model learns the OTU composition for each of a fixed number of source communities, where all samples for a given source must share a single mixture of OTUs, and then uses this information to predict how much each source might contaminate a given test sample. Holmes *et al*. [13] employed an approach similar to latent Dirichlet allocation (LDA) to model microbiome composition. Their model relaxes the assumption that, when learning about a microbiome structure, all samples for a given feature will share a single mixture of OTUs. However, their model does not provide a means of assessing the structure of the microbiome in terms of readily interpretable parts. In this study, we describe a novel model (called Bayesian inference of microbial communities (BioMiCo)) that is intended to facilitate interpretation of a community structure in light of user-defined feature labels. Ours is a hierarchical model that can be used to simultaneously learn how assemblages of OTUs contribute to microbiome structure and how multiple assemblages might be related to the known features of the samples.

BioMiCo can be applied to cross-sectional or serially sampled data. Serially sampled data is desirable because it can help to rule out cases where microbial communities have merely accumulated *post aut propter* differences. More critically, serially sampled data are especially valuable for statistical validation of the model; by dividing the data into two independent parts (for example, samples taken during different time periods), the predicative accuracy of the model can be directly measured at different time points. Separate phases of the analysis (training and testing) are applied to the independent datasets. In the training phase, the model is applied to only one part of the data and supplied with labels for the features of interest. This is the phase in which the model learns how to use microbiome structures (assemblages of OTUs) to predict the features of interest. In the testing phase, the model is applied to a different part of the data, but it is not supplied with the feature labels; that is, it must predict the “hidden” labels according to what it has previously learned about microbiome structure. Knowledge of the hidden labels can thus be used to quantify the predictive accuracy of the model for data collected at different time points. Note that supervised methods do not require serially sampled data. Multiple samples can be taken at a single time point and divided into two parts for training and testing. However, we chose to apply BioMiCo to three serially sampled datasets. The first dataset is from a study of microbiome variation in two humans at four body sites over the course of 6 to 15 months. The second is from a study of the human vaginal microbiome over the course of 4 months. The third is from a study of temperate coastal marine communities sampled for over 6 years. We use these data to illustrate how BioMiCo can be employed to investigate community structure with respect to specific features of interest, and we explicitly evaluate the accuracy of the model to make predictions at different points in time.

## Methods

### Overview of the analytical framework

We assume that each sample contains information about the abundance of microorganisms within the sampled environment, which will typically be derived from environmental DNA (16S or whole genome sequencing (WGS)). Microorganisms within a sample are expected to show a range of interactions, from very little inter-dependence to obligate symbiosis [3]. Here, we refer to a set of co-occurring OTUs resolved by the model as an assemblage, where the ecological interactions among the members of the assemblage are treated as unknown. However, the microbial associations that are detected with the model can be the starting points for detecting ecological interactions. Indeed, the most direct test for microbial interactions is to experimentally disturb the microbiota and assess the recovery of the same pattern of OTU associations as originally resolved by the model. We follow Boon *et al*. [3] by using the term community to refer to a collection of OTUs that are explicitly assumed, or known, to have a high degree of ecological interaction.

Inference about assemblages under BioMiCo can be based on taxonomic units such as OTUs or on functional units such as the Kyoto Encyclopedia of Genes and Genomes (KEGG) Orthology numbers. In all three datasets used in this study, we modeled microbiome structure as statistical mixtures of OTU assemblages. The model can then be used to explain any factor label in terms of several assemblages. Factor labels used in this study included the identity of a human host (for human gut microbiomes), Nugent score (for human vaginal microbiomes), and season of the year (for a coastal marine microbiome). In many settings, the factor labels are chosen because they are believed to indicate some degree of microbial community structure or function. The idea that communities are composed of partially overlapping assemblages of organisms having variable types of ecological interaction is not new to ecology [15] or to microbiology [16,17]. However, this model is the first to offer a probabilistic framework to discern their structural components according to environmental DNA.

Microbiome samples often represent mixtures of different microbial communities. Sometimes, this is because it is more convenient to sample mixtures (for example, stool samples are comprised of mixtures of epithelium and luminal niche communities [18], but they are much more easily obtained than epithelial biopsies). Sometimes, samples will be mixtures of communities because we simply do not have sufficient knowledge to precisely target the community of interest (for example, at certain times of the year, a marine community might be stratified or mixed depending on a variety of physical and chemical factors [19]). Because we cannot assume that microbiome samples will always correspond to a single community, we consider each sample as a potential mixture of OTUs from one or more communities. In some cases, it is even desirable to collect and train on samples that represent communities that are mixed to different degrees. Consider the possibility that a difference in microbiome metabolic function could contribute to the intensity of a disease phenotype. In such cases, by training on samples having various mixtures of different communities, the model can learn if a patient’s health status might depend on the degree to which functional and dysfunctional microbial communities contribute to his/her personal microbiome.

The model is supervised, using a portion of the data reserved for “training” to learn which assemblages are associated with a set of pre-selected features of interest. Within BioMiCo, features of interest are called “factor labels,” and the specific value for a given sample is called a “factor value.” Labels can represent generalized factors of interest (for example, season of the year, ethnicity, health status) or specific attributes of a sample (for example, the identity of the human host in serially sampled data). Users can specify values for multiple factor labels for a single sample by providing unique indicator variables for values coming from more than one label. The training set of samples is used to learn the mixture weights for both (*i*) the mixing of OTUs within assemblages and (*ii*) the mixing of assemblages within samples according to factor values shared across multiple samples. The remaining samples are called the “test set.” Based on the mixture weights learned from the training samples, we compute the posterior probability that a test sample originated from a microbiome having any of the factor values that the model was trained on. If discrete assignment according to factor values is desired, each test sample can then be classified according to the maximum posterior probability. Since in this study the true label value for each test sample is known, accuracy is measured as the percent of the factor values that are correctly predicted for test samples.

### BioMiCo: a hierarchical mixed-membership model

We model each microbiome sample as a mixture of OTUs from one or more communities by using *K* pre-specified factors values (corresponding to one or more labels) as putative mixture components. For simplicity, hereafter, we refer to these mixture components as “factors.” While the value of *K* is fixed within the model, the relative importance of the factors is permitted to differ between samples. The relative contribution of each factor to the *n*th sample is modeled through the latent variable *π*_*n*_. Furthermore, we only require that some subset of this set of known factors is contributing to a given sample. This is different from assuming that every sample is a mixture from all the factors of interest. If this sample can be assumed, *a priori*, to reflect the contribution of as many as, say, four factors, then *π*_*n*_ is a probability vector of four non-zero values summing to one. The probability values for all other non-contributing factors are set to zero. Because we will not know the community structure of the sample, the *π*_*n*_ variables are inferred from the data. We assume a symmetric Dirichlet prior on *π*_*n*_:

$$ {\pi}_n\sim \mathrm{Dirichlet}\left({\alpha}_{\pi}\right) $$

A symmetric Dirichlet is appropriate because we have no prior preference for any of the factors.

Next, we assume that the OTUs that characterize a factor are comprised of a fixed number of OTU assemblages (*L*). Therefore, the model differentiates factors according to their unique mixture of assemblages. The mixture for the *k*th factor is modeled by a vector of *L* mixing probabilities, *θ*_*k*_, that sum to one. Thus, there will be *K* vectors of *L* mixing probability values. The element *θ*_*kl*_ in a *K* × *L* matrix, *θ*, represents the relative contribution of assemblage *l* to factor *k*. We assume a symmetric Dirichlet prior on rows of *θ* because we have no prior knowledge to favor particular assemblages:

$$ {\theta}_k\sim \mathrm{Dirichlet}\left({\alpha}_{\theta}\right)\ \mathrm{f}\mathrm{o}\mathrm{r}\ k=1\cdots K $$

Finally, we assume that each assemblage is comprised of a mixture of *T* different OTUs. The contribution of different OTUs to the *l*th assemblage is modeled by a vector of *T* mixing probabilities, *ϕ*_*l*_, with values summing to one. Given *L* assemblages, there will be *L* probability vectors of length *T* representing OTU mixing probabilities. Thus, element *ϕ*_*li*_ in an *L* × *T* matrix *ϕ* represents the relative contribution of OTU *i* in assemblage *l*. We also assume symmetric Dirichlet prior on rows of *ϕ*:

$$ {\phi}_l\sim \mathrm{Dirichlet}\left({\alpha}_{\phi}\right)\kern0.5em \mathrm{f}\mathrm{o}\mathrm{r}\kern0.5em l\kern0.5em =\kern0.5em 1\kern0.5em \cdots \kern0.5em L $$

**Figure 1:**
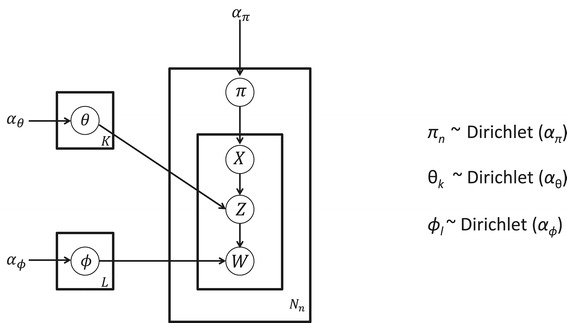
**Plate diagram of the mixed-membership model for BioMiCo.** *π* is the probability distribution on the possible source environments, *X* represents the environments, *Z* represents the assemblages, *W* represents the OTUs, *θ* is the prior distribution of assemblages in environments, and *ϕ* is the prior distribution of OTUs in assemblages. The variable *α* represents the concentration parameters for prior distributions, with *α*_*θ*_ being the concentration parameter for the prior on the distribution of assemblages in environments, *α*_*ϕ*_ the concentration parameter for the prior on the distribution of OTUs in assemblages, and *α*_*π*_ the concentration parameter for the prior on the distribution of environments. *N* is the number of samples in a dataset, and *N*_*n*_ is the number of OTUs in sample *n. K* is the number of environments, and *L* is the number of assemblages.

We use symmetric Dirichlet because we have no prior knowledge to favor a particular OTU in an assemblage. Thus, the model is used to differentiate assemblages according to their unique mixture OTUs. A plate diagram of the model is shown in Figure 1.

### Model inference

The model is used to learn the mixing of OTUs within assemblages and the mixing of assemblages that characterize a factor value. Let the factor and assemblage assignments for OTU *i* in sample *n* be denoted by *X*_*ni*_ and *Z*_*ni*_, respectively. Note that *Z*_*ni*_ is a value between one and *L* (number of assemblages) and *X*_*ni*_ is a value between one and *K* (number of factors). *W*_*ni*_ is the OTU *i* in sample *n* and has a value between one and *T*. For inference, we integrate out all other latent variables and sample from the posterior distribution of the assemblage and factor assignments (*Z* and *X*) for each OTU given the data. We use collapsed Gibbs sampling [20] for posterior inference. Given a set of variables, in each state of the Markov chain, some (one or more) variables are sampled conditioned on the current values of all other variables and the data in some order.

The complete likelihood of the data given the hyper-parameters of the prior distributions in our model can be written as follows:

$$ P\left(W,Z,X,\phi, \theta, \pi \Big|{\alpha}_{\phi },{\alpha}_{\theta },{\alpha}_{\pi}\right)= $$

$$ P\left(W\Big|Z,\phi \right)\ P\left(Z\Big|X,\theta \right)\ P\left(X\Big|\pi \right)\ P\left(\phi \Big|{\alpha}_{\phi}\right)\ P\left(\theta \Big|{\alpha}_{\theta}\right)\ P\left(\pi \Big|{\alpha}_{\pi}\right)= $$

$$ {\displaystyle \prod_{n=1}^N}\ {\displaystyle \prod_{i=1}^{N_n}}P\left({W}_{ni}\Big|{\phi}_{Z_{ni}}\right) \times {\displaystyle \prod_{n=1}^N}\ {\displaystyle \prod_{i=1}^{N_n}}P\left({Z}_{ni}\Big|{\theta}_{X_{ni}}\right) \times $$

$$ {\displaystyle \prod_{n=1}^N}\ {\displaystyle \prod_{i=1}^{N_n}}P\left({X}_{ni}\Big|{\pi}_n\right)\ \mathrm{P}\left(\phi \Big|{\alpha}_{\phi}\right)\mathrm{P}\left(\theta \Big|{\alpha}_{\theta}\right)\mathrm{P}\left(\pi \Big|{\alpha}_{\pi}\right) $$

where *Z*, *X*, *θ*, *ϕ*, *π* are the set of latent variables in our model, *N* is the number of samples in a dataset, and *N*_*n*_ is the number of OTUs in sample *n*. For posterior inference, we want to sample from the posterior distribution of latent variables given the data:

$$ P\left(Z,X,\theta, \phi, \pi \Big|W,{\alpha}_{\phi },{\alpha}_{\pi },{\alpha}_{\theta}\right) $$

Because we are interested in sampling from the assemblage (*Z*) and factor (*X*) assignments for each OTU given the data, we integrate out the other latent variables (*θ*, *ϕ*, *π*) and sample from the posterior distributions of *Z* and *X*, or *P*(*Z*, *X*|*W*, *α*_*ϕ*_, *α*_*π*_, *α*_*θ*_). This posterior distribution can be derived analytically for our model [see Additional file 1]. We use Gibbs sampling for drawing samples from this distribution.

At each iteration of the Gibbs sampling, we repeat the following in a random order. For each OTU in each sample, we draw the assemblage and factor assignment (*Z*_*ni*_ and *X*_*ni*_, respectively) of this OTU given the current assemblage and factor assignments of all other OTUs in this and every other sample. Let *Z*_−*ni*_ represent the assemblage assignment for all OTUs in all samples except only OTU *i* in microbiome sample *n*. And, let *X*_−*ni*_ represent the factor assignment for all OTUs in all samples except only OTU *i* in microbiome sample *n*. Each of the individual terms for the conditional distribution can be analytically derived:

$$ P\left({X}_{ni}=k,{Z}_{ni}=l\Big|{X}_{-ni},{Z}_{-ni},W,{\alpha}_{\phi },{\alpha}_{\pi },{\alpha}_{\theta}\right)= $$

$$ \frac{C_{w_{ni}l} + {\alpha}_{\varphi }}{{\displaystyle {\sum}_{w^{,}}}\left({C}_{w{\hbox{'}}_{ni}l} + {\alpha}_{\varphi}\right)}\times \frac{C_{kl} + {\alpha}_{\theta }}{{\displaystyle {\sum}_{l^{\hbox{'}}}}\left({C}_{k{l}^{\hbox{'}}} + {\alpha}_{\theta}\right)}\times \frac{C_{nk} + {\alpha}_{\pi }}{{\displaystyle {\sum}_{k^{,}}}\left({C}_{nk\hbox{'}} + {\alpha}_{\pi}\right)} $$

where ${C}_{w_{ni}l}$ is the number of times OTU *i* in sample *n* is drawn from assemblage *l. C*_*kl*_ is the number of times an OTU is drawn from assemblage *l* contributing to factor *k. C*_*nk*_ is the number of times an OTU in sample *n* is drawn from factor *k*. Solutions for the individual terms of the conditional distribution are provided in Additional file 1. From the above equation, a value is calculated for each possible combination of values for *X*_*ni*_ and *Z*_*ni*_. Note that *X*_*ni*_ can take only factor assignments that are known to be contributing to sample *n*. These values are then normalized and used to draw new assignment values for *X*_*ni*_ and *Z*_*ni*_ which are immediately updated for use in the next iteration. In this study, we also employed a Metropolis-within-Gibbs sampling scheme to learn the hyper-parameters (*α*_*θ*_, *α*_*ϕ*_, and *α*_*π*_) from the data. Metropolis-Hastings updates were used to resample new values for hyper-parameters once all the other parameters of the model have been sampled. Analyses of all datasets were initialized with hyper-parameter values of 0.01. In this study, the first 200 iterations of the Markov chain Monte Carlo (MCMC) for the training phase were considered “burn-in” and were discarded. Following the burn-in, the MCMC was run for a minimum of 2,000 iterations, and samples were retained every 100 iterations. Chains were run multiple times, and if the posterior distribution of assemblages was not concordant among runs, the chains were run longer.

### Predicting the contribution of different factors to an unlabeled sample

This model can be used to predict the factor values for a sample for which we do not have factor assignments. Labels are chosen by the user, and they can represent specific factors of interest (for example, healthy vs. diseased state) or, more generally, the physical aspects of an environment (for example, different concentrations of a limiting nutrient). The model is first trained on data where each sample is assigned to a possibly distinct known subset from the set of all available factors. The trained model is then applied to test data to *predict* factor contributions for each sample. Known features of samples can be intentionally hidden from the model for the purpose of testing its performance.

We want to sample the posterior distribution of factor assignments given the OTU distribution of the test data by using what the model has learned from the training data; that is, *P*(*X*test|*X*train, *Z*train, *W*, *α*_*ϕ*_, *α*_*π*_, *α*_*θ*_). To achieve this, we sample from posterior distribution of *X*test and *Z*test jointly, that is, *P*(*X*test, *Z*test|*X*train, *Z*train, *W*, *α*_*ϕ*_, *α*_*π*_, *α*_*θ*_), and marginalize over the assemblage assignments to obtain the posterior probability of each factor assignment. In this analytical phase (testing), Gibbs sampling is very similar to the training process. The difference is that we do not need to iterate over the samples in the training set; we carry forward the count variables ${C}_{w_{ni}l}$ and *C*_*kl*_ from the training phase, and we do not need to run the MCMC for as many iterations. The first 50 iterations of the testing phase were discarded as “burn-in,” and the MCMC was run for an additional 1,000 iterations with samples retained every 50 iterations. In all datasets examined in this study, the number of factor labels (*K*) is determined by the research question, and the number of assemblages (*L*) was set to 100.

### Implementation

Our implementation of the model and inference algorithm is called BioMiCo, and the source code (C++ and R) is available at http://sourceforge.net/projects/biomico. Being a generative model, we simulated a microbiome sample for the purpose of testing the inference algorithm. See Additional file 2 for an overview of the generating process. We simulated data over a very wide range of conditions (504 scenarios, each comprised of 200 microbiome samples) and used it to (*i*) verify that our training phase can recover the parameter values used to generate structured microbiome and (*ii*) investigate what conditions represent easy and hard inference problems for the testing phase. Results confirmed the reliability of the inference algorithm in the training phase and demonstrated that reliable inference of factor labels is possible over a very wide range of conditions. Additional file 2 contains a full description of the simulation design and presents the outcomes of the simulation study.

## Results and Discussion

### Dataset 1: assessing temporally stable microbial assemblages within the human microbiome

We applied our mixture modeling approach to a detailed investigation of temporal microbiome variation, which entailed long-term sampling of two human individuals at four body sites (gut, tongue, right and left palm) over 396 time points [1]. As both individuals in this study were healthy, there were no clinically significant factors to train on. Although temporally stable strains have been detected within other longitudinal studies of the human gut [4,21,22], temporal stability could not be detected in this study [1]. This makes the Caporaso *et al.* [1] dataset an especially rigorous test of our approach, which treats OTUs as potential members of assemblages rather than as independent units of diversity. By combining information from many OTUs within an assemblage (including OTUs having low abundances), we anticipate improved power to detect a temporally stable signature in those data. Further, by training over a small time period (for example, 1 month) and testing the provenance of the samples taken at other times, we can measure the reliability of predictions made according to assemblage information for a long window of time.

**Figure 2:**
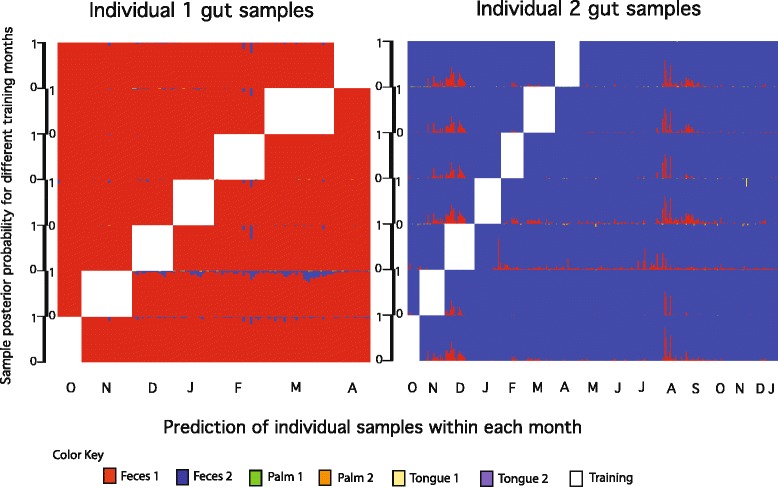
**Prediction of both human host identity and body site according to maximum posterior probability.** Samples were from the gut microbiomes of two individuals collected over 7 and 16 months (individuals 1 and 2, respectively) [1]. The white blocks within the plots are the months that were used to train the model. Every row corresponds to the results obtained from a different training month. The height for each row corresponds to the posterior probability scale of 0 to 1. The posterior probabilities for the palm and tongue samples from the same study can be found in Additional file 3.

**Figure 3:**
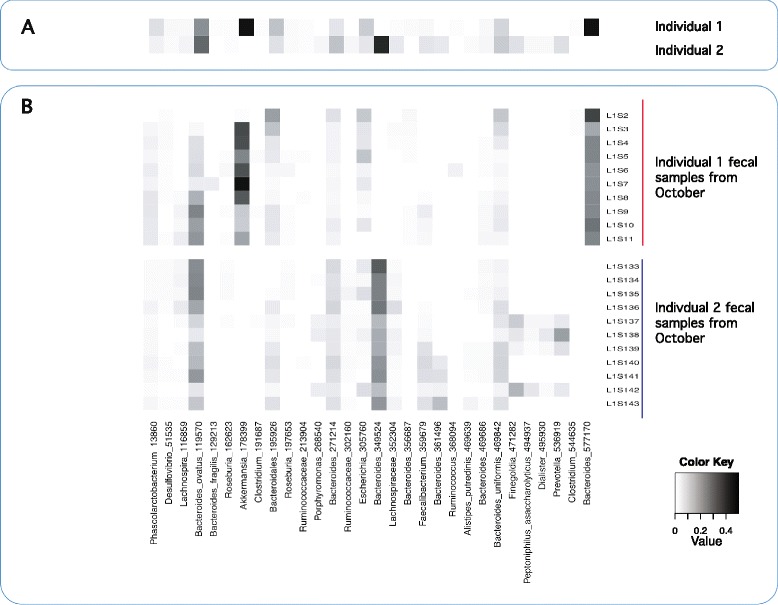
**Heatmap showing the contributions of particular OTUs that characterize fecal samples of individuals 1 and 2. (A)** Posterior probabilities for OTUs (same labels as in **(B)**) as determined by the model. **(B)** Empirical abundance of OTUs in samples collected in October 2009 and used to train the data (sample IDs listed on the right are from Caporaso *et al.* [1]). Note that 41 OTUs accounted for 95% of the posterior density for individual 1, and 86 OTUs accounted for 95% of the posterior density of individual 2. For clarity, we present only the 20 OTUs that have the highest posterior probability in each individual. For individual 1, the top 20 OTUs account for 90% of the posterior density. For individual 2, the top 20 OTUs account for 79% of the posterior density. The top 20 OTUs can be thought of as the “predominant” OTUs for each individual.

The original set of OTU counts generated by Caporaso *et al*. [1] was filtered to remove all singletons (that is, OTUs observed in only one sample). Our model can be run on data that includes singletons, but they were excluded because they increase computational costs without providing a useful signal (and could potentially dilute signal strength for the assemblage membership of other OTUs). Filtering yielded a matrix of 1,967 samples (rows) having 15,685 OTUs (columns). This matrix represented the samples obtained from 396 time points for two human hosts over four body sites; each sample was labeled according to both host and body site (thus, *K* = 2 × 4 = 8 in this model). As was already reported [1], we found pronounced variability in microbiota from both subjects across months, weeks, and even days. Also, like the original study, samples from different body sites were easily distinguished throughout the sampling interval (Figure 2 and Additional file 3). However, we also uncovered evidence of a hierarchal structure in the form of assemblages of OTUs; the values of both *α*_*ϕ*_ and *α*_*θ*_ estimated by using Metropolis-Hastings when training on seven different months were substantially less than 1.0 (*α*_*ϕ*_: mean = 0.006, min = 0.005, max = 0.007; *α*_*θ*_: mean = 0.021, min = 0.012, max = 0.026). Further, the mixture weights revealed (*i*) some OTUs had similar, and temporally stable, mixture weights in both individuals (for example, *Bacteriodes ovatus* 119570 in Figure 3), (*ii*) some OTUs had consistently different mixture weights between individuals (for example, *Bacteriodes* 577170 in Figure 3), and (*iii*) most OTUs had no temporal stability. For this dataset, assemblages are composed of OTUs that tend to have the same co-occurrence pattern across serially sampled microbiomes; thus, they can represent a temporally stable signature within these data. Figure 3A shows OTUs from assemblages that are characteristic of fecal samples from individuals 1 and 2; note that they are composed of OTUs with varying degrees of abundance yet follow the same co-occurrence pattern.

Because the individual-specific mixture weights (that is, pattern *ii* above) represent a central tendency in OTU composition within the human microbiota, this signal could serve as the basis for making predictions through time. To test the accuracy of a prediction based on such a signal, we attempted to predict the most comprehensive feature of a sample, the identity of the human host, based on mixing probabilities learned from samples taken weeks and even months earlier. This approach yielded very high-classification accuracy when using the signal contained within the gut (97%) and the tongue (87%) microbiomes. The signal persists over the full duration of the study. Re-training on different months yielded accuracy between 98.6% and 99.3% when using gut samples and 85% to 93% when using tongue samples. Prediction was more challenging when using the signal contained within palm samples (40% to 75% accuracy over the study). However, samples from the human palm pose a special challenge because they are exposed to a wide range of microbial environments over days, weeks, and years.

Initially, we identified a number of outliers for several gut samples, that is, we detected high-posterior probabilities for body sites that were not the site of provenance for the samples in question. This was surprising since samples from the gut microbiome generally had strong individual-specific microbiome signatures. Subsequently, we discovered that these outlier samples had been mislabeled and were excluded from the original study [1]. Exclusion of the mislabeled samples from our study and retraining did not substantially alter classification accuracy. This illustrates that our modeling framework is powerful, even when labeling errors are included in the training set [see Additional file 4].

Some OTUs within the gut microbiome appear to be highly discriminatory; that is, they have a high abundance in one individual and a low abundance in the other. To test that the model is using the co-occurrence information of the OTUs in an assemblage, and not just the frequency differences of those few high-abundance OTUs in the gut, we removed the OTUs with an average relative abundance of greater than 1% in the fecal samples (by setting the raw counts to zero for those OTUs in the fecal test data), and we re-predicted each sample’s host. This led to the exclusion of 14 OTUs from individual 1 and 16 OTUs from individual 2. In those cases where testing was possible, classification accuracy was between 81% and 100%. There were two sets of training data where testing was not possible because in those 2 months (October and November) the training data for individual 1 was completely dominated by the 14 OTUs removed; thus, there was no basis for classification of individual 1 according to those training data. These results indicate that co-occurrence information for low-abundance OTUs contributes to the ability to make predictions at different time points.

Recall that previous studies were unable to detect any temporal stability within these data. In contrast, we found that some OTUs maintained host-specific co-occurrence patterns in the face of dramatic temporal variation in community composition. Our focus on assemblages (which includes the low-abundance OTUs), and our implementation of a supervised framework, made this possible. Note that we do not anticipate that our model will normally be used to identify hosts within serially sampled datasets, as they will be known with certainty. We also expect that sampling additional human hosts will likely reveal some sharing of assemblages (for example, among family members). Our results, however, indicate that (*i*) there exists temporally stable assemblages within human-associated communities and (*ii*) reliable predictions can be made at future time points according to information about microbial assemblages obtained from training data. If links can be made between microbial assemblages and status labels for human health (for example, obese, frail, Crohn’s disease, remission), the value of microbiomics to personalized healthcare might be further enhanced.

### Dataset 2: assessing microbial communities in women with no symptoms of bacterial vaginosis and high Nugent scores

Bacterial vaginosis (BV) is a condition that predisposes women to greater risk of sexually transmitted disease and adverse pregnancy outcomes [23,24]. Nugent *et al*. [25] described a method of scoring a vaginal smear according to the relative abundance of selected microbial lineages, and this score is commonly used in research settings to indicate BV. A score between 7 and 10 is considered indicative of bacterial vaginosis, although a score ≥4 in conjunction with “bacteria covered” clue cells within the smear is also considered consistent with BV. Based on a comprehensive study of temporal dynamics in vaginal microbiomes of asymptomatic women, Gajer *et al*. [26] suggested that the notion of a healthy vaginal microbiome might need to be expanded to include the possibility of high Nugent scores. Some women’s Nugent score varied substantially over the course of their study, while others had a long-term tendency to score high; yet none of the women reported any symptoms of BV. Given these temporal dynamics, we wanted to determine if our model could identify a community signature consistent with elevated Nugent scores in asymptomatic women. We also wanted to investigate the possibility of additional structure; that is, that there might be some temporally stable differences among women who persistently score high on the Nugent scale.

We applied our modeling framework to the data of Gajer *et al*. [26], which was collected twice weekly from 32 reproductive-age women over a 16-week period. As menses can have a dramatic impact on the vaginal community [26], we excluded samples taken during menses. We also excluded samples for which there was no Nugent score. This yielded a matrix of 715 samples (rows) having 330 OTUs (columns). We used a common scheme for discretizing Nugent scores [25,26] and assigned all samples to one of two categories: “normal” (low score: 0 to 3) and “elevated” (intermediate score: 4 to 6; high score: 7 to 10) Nugent scores. Thus, for this model, *K* = 2. We then randomly assigned two thirds of the samples in each category to a training set and trained the model on those data. The remaining one third of the samples was used to test the accuracy of using OTU co-occurrence patterns to predict an elevated Nugent score. This procedure was replicated five times.

**Figure 4:**
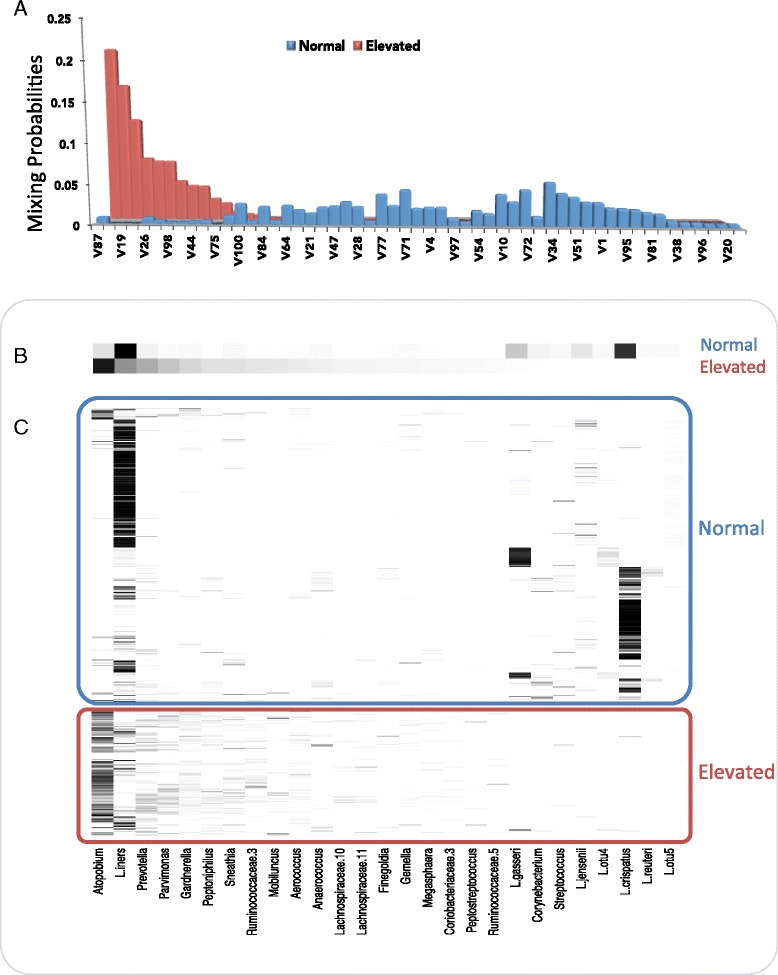
**Posterior distribution and composition of microbial assemblages with respect to “normal” and “elevated” Nugent scores in asymptomatic human females.** Distributions were inferred from the vaginal microbiomes of 32 individuals collected over a 16-week period [26]. **(A)** Mixing probabilities for the assemblages comprising 95% of the posterior distribution for “normal” and “elevated” Nugent scores. “Normal” was defined by a Nugent score of 0 to 3, and 47 assemblages were responsible for 95% of their posterior distribution. “Elevated” was defined by a Nugent score >4, and 12 assemblages were responsible for 95% of their posterior distribution. As five assemblages were shared between these two distributions, there are a total of 54 assemblages in this plot. **(B)** Relative magnitude of the mixing probabilities of 27 OTUs (identity is indicated along the *x*-axis of part **(C)** marginalized over the 54 assemblages in part **(A)**. Note that 14 OTUs accounted for 95% of the posterior density (PD) of the “normal” Nugent score category and 19 OTUs accounted for 95% of the PD of the “elevated” Nugent score category. As six OTUs were shared between these two distributions, there are mixing probabilities for a total of 27 unique OTUs in this plot. **(C)** Empirical relative abundance of the 27 OTUs in the 484 training samples. OTU identity is given along the *x*-axis. Each row represents an individual sample.

Figure 4 summarizes the structure of the assemblages learned by the model and their mixing probabilities with respect to “normal” and “elevated” Nugent scores. The difference between assemblage distributions (Figure 4A) is due to differences in the depth of community structure. The group of individuals having a “normal” Nugent score had a relatively flattened distribution of highly sparse assemblages. Sparse assemblages are characterized by just a very few (in this case one to three) OTUs with non-trivial mixing probabilities. The flat assemblage distribution reflects the contribution of many low-abundance OTUs to the “normal” label, as well as the tendency of *Lactobacillus iners* and *Lactobacillus crispatus* to co-occur with different low-abundance OTUs in different individuals. The group of individuals having “elevated” Nugent scores had a deeper community structure, that is, more complex co-occurrence patterns concentrated in fewer assemblages, yielding a much more skewed assemblage distribution. As the Nugent score is based on the premise that Lactobacilli decrease the score, and that *Gardnerella* or *Bacteroides* spp. or curved gram variable rods increase the score, it is not surprising that this signal is contained in the OTU assemblages (Figure 4). However, the OTU composition of the assemblages with the highest mixing probabilities provides additional information about individuals with elevated Nugent scores; they represent the central tendency of co-occurrence relationships over all the training samples having an “elevated” Nugent score. To the extent that these patterns have temporal stability, they can be used to make predictions about unlabeled data.

Patterns of OTU co-occurrence were informative for making predictions about the test data. Mean classification accuracy over the five random assignments of microbiome samples to the training partitions was 90% (range: 88% to 93%). Interestingly, a large fraction of classification errors were associated with outlier Nugent scores; these are cases where an individual has a consistent time series of “elevated” or “normal” Nugent scores with a single outlier score within this time series. Approximately 50% of the misclassification errors were for this type of outlier; thus, overall classification is improved when we attempted to predict an individual’s median Nugent score (92% accuracy; range: 90% to 95%) rather than their sample-specific scores. Note that prediction of median scores for seven individuals was a little less accurate than their sample-specific predictions. Remarkably, the prediction of sample-specific Nugent scores was reliable when an individual switched Nugent score categories as long as that switch persisted for a few samples. As the model does not explicitly include temporal autocorrelation, this finding hints at potentially interesting lurking variables that influence microbiome stability over different time scales.

We also investigated if predictions for these data exploited the assemblage information and not just the frequency differences of a few high-abundance OTUs. We removed the OTUs having >1% contribution to the vaginal microbiome samples. This led to the exclusion of seven OTUs from the “normal” score category and 11 OTUs from the “elevated” score category. Classification based on just the low-abundance OTUs was largely unaffected by this data reduction; mean accuracy remained at 90% (range: 89% to 93%). These results verify that the model was able to capture community-wide signatures associated with elevated Nugent scores. To obtain a Nugent score, an experienced microbiologist must assess bacterial cell morphology within a pap smear; we suggest that moving toward an OTU-derived scoring system could mitigate this difficulty. Further, by training on additional variables, model-derived scoring systems could be developed for more narrowly defined features of interest.

Next, we investigated the possibility of community diversity among individuals within the group of asymptomatic women with elevated Nugent scores. We used two analytical approaches. First, we simply restricted our analyses to the ten women having a median Nugent score ≥4 and tested for individual specific OTU assemblages (*K* = 10). Second, we trained a model on the full data where each sample was labeled with respect to both the Nugent score category and the host identity. In this design, the maximum value of *K* = 2 × 32 = 64; however, *K* = 53 in the model because some individuals always belonged to a single Nugent score category. For each approach, we carried out five replications where we randomly assigned two thirds of the samples to a training set and used the remaining one third of the samples as a test set. We achieved a remarkably high accuracy (>83%) in predicting the provenance of those samples collected from individuals having a median Nugent score ≥7 (Table 1). We had only moderate success at predicting host provenance given intermediate Nugent scores and even less for individuals having low Nugent scores (Table 1). These results indicate that women with the highest Nugent scores harbored additional microbial assemblages, and these differences had a remarkable degree of temporal stability over the course of the study. This suggests directions for future research. Again, we do not expect that future studies will typically train a model according to individual host identity. We do suggest that temporally stable differences among women with high Nugent scores could be related to long-term differences in their status (for example, some women might remain asymptomatic, others might experience limited clinical symptoms, and others might develop recurrent BV). By combining longitudinal studies of both asymptotic and BV cases (for example, [27]), and training on additional factor labels (pH, discharge, clue cells, persistent BV, remission, relapse), supervised analyses under BioMiCo could lead to a more refined understanding of the relationship between community structure and an individual’s manifestation of BV. BioMiCo is not intended as a substitute for other statistical methods [11,28]; rather, it should be viewed as complementary to the methods which are already used to investigate community structure and track their changes over time (for example, [26,29]).

**Table 1:** **Percent correct classification of host provenance according to assemblages in the vaginal microbiomes of asymptomatic women**

	**Median Nugent score category**		
	**“Elevated score”**		**“Normal score”**
**Analysis**	**High (≥7)**	**Intermediate (7–4)**	**Low (<4)**
Approach 1	90	54	NA
Approach 2	83	43	38

Percent correct classification is based on five replications of random assignment of two thirds of the samples to a training set and using the remaining one third for testing host provenance. Approach 1 is an analysis of individuals with elevated scores and training only on host provenance. Approach 2 is an analysis of all individuals and training on samples labeled according to both host provenance and Nugent score.

### Dataset 3: assessing seasonality in coastal ocean bacterial communities

Bacterial communities in the ocean are highly diverse [30] and variable across space and time [19,31]. Since community variability is often associated with changes in ecosystem-level processes, the identification of assemblages of possibly interacting bacterial taxa - and the description of how these assemblages change in time - can contribute to our understanding of how marine ecosystems respond to environmental change. The detection of time-dependent co-occurrence patterns among microbial OTUs has been widely employed in time-series studies of marine microbial communities [32]. These studies have shown that ocean bacterial communities exhibit repeating patterns such as seasonal succession and annual reassembly [33], and that seasonality can often be linked to environmental conditions and resource availability [2,34]. Given these temporal dynamics, we aimed to determine if our model could be applied to ocean time-series data to identify community assemblages that are characteristic of different seasons. Once trained, we also wanted to investigate whether or not the predictive component of the model could provide insight into when and how rapidly bacterioplankton community succession occurs in the ocean.

**Figure 5:**
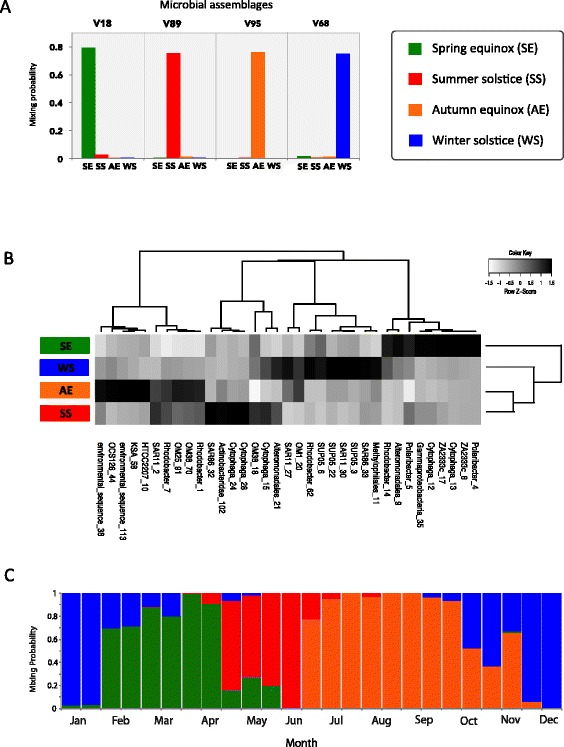
**Posterior distribution and composition of seasonal assemblages in a coastal bacterial community. (A)** Mixing probabilities for microbial assemblages at four distinct seasonal time points. Colors used to define seasons were spring equinox (SE) - green; summer solstice (SS) - red; autumn equinox (AE) - orange; and winter solstice (WS) - blue. Samples were from 24 surface (1 m) collections taken from 2005 to 2010 in the Bedford Basin, a coastal inlet of the temperate northwest Atlantic Ocean [35]. **(B)** Hierarchical clustering of OTU-mixing probabilities from the four seasonal assemblages in part **(A)**. For these assemblages, a very large number of OTUs contribute 95% of the posterior density (PD), but a small subset contributes a disproportionately large amount of that density. We refer to this influential subset as the predominant OTUs and define them according to the inflection point in their posterior OTU distribution. For clarity, we clustered only the predominant OTUs. For SE, there were 15 predominant OTUs (75% of PD). For SS, there were 15 predominant OTUs (73% of PD). For AE, there were 17 predominant OTUs (62% of PD). For WS, there were 19 predominant OTUs (66% of PD). **(C)** Model-based predictions for the high-resolution time series collected biweekly from January to December in 2009.

As proof of principle, we applied our modeling framework to the data of El-Swais *et al*. [35], which consisted of a set of OTUs from bacterial communities in the Bedford Basin, a coastal inlet of the temperate northwest Atlantic Ocean sampled over a 6-year period. We trained the model on 24 samples collected from 2005 to 2010; each sample was labeled according to four distinct seasonal time points (thus, *K* = 4 in this model). The factor values for training consisted of the spring equinox (SE), summer solstice (SS), autumn equinox (AE), and winter solstice (WS). The training phase identified four highly informative assemblages, each contributing a high posterior mixing probability to one of the four seasons (Figure 5A). Hence, as expected, samples from different seasons were clearly distinguishable from each other. More interestingly, seasonal differences in the mixing probabilities of OTUs revealed putative patterns of ecological interaction between taxa. A number of OTUs had high-mixture weights for only one assemblage, which is indicative of seasonal specificity. For example, *Polaribacter*, *Cytophaga*, and Alteromonadales OTUs were important members of the SE assemblage (Figure 4B), which supports earlier studies reporting that these taxa are often associated with the consumption of organic matter from spring phytoplankton blooms [36,37]. During the summer, the Bedford Basin surface waters are characterized by low-nutrient availability [38]. As such, it follows that OTUs from the SAR11 and SAR86 clades were principal members of the SS assemblage (Figure 4B), in agreement with the known oligotrophic nature of these marine bacteria [39,40]. These results demonstrate that even with only a few training samples (six per each season), the model can identify ecological structure in the data. Readers should consult El-Swais *et al*. [35] for additional details about the temporal variability of bacteria in the Bedford Basin.

Once the model training was complete and the seasonal assemblages identified, we tested how well the model predicted an additional 25 samples collected at biweekly intervals in 2009. The transitions between assemblages observed within these data, as inferred by the temporal changes in the mixing probabilities, reflected the seasonal cycle of community succession in 2009 (Figure 4C). Most intriguingly, the timing of the mixing probability transitions, and the time span that a certain assemblage contributed to a sample over the year, was strongly linked to certain events known to occur in the Bedford Basin. For example, a shift from a winter assemblage to a dominant spring assemblage occurred in early February, which corresponded with the onset of the spring phytoplankton bloom [35]. The spring assemblage continued to dominate through to the end of April, which marked the end of the spring bloom, and was then succeeded by the summer assemblage. In these cases, the model accurately predicted the timing of known ecosystem-level events based on the OTU composition of samples. However, the model also provided additional insight into bacterial dynamics in the ocean. A clear example of this is the “return” of the autumn assemblage in late November, which was a striking observation since we expected a steady transition from the autumn assemblage to the winter assemblage from October through December. As it turns out, a short phytoplankton bloom occurred in November, and the return of the autumn assemblage is presumably a response in the bacterial community to this episodic event. Collectively, these results demonstrate how a predictive model can be used to track the rate and timing of community succession and predict known ecosystem-level events in the marine environment.

## Conclusions

We have presented a novel analytical framework for the probabilistic modeling of a microbial community structure. The approach is based on a model that has several unique features, but foremost is the use of a hierarchical structure for modeling microbiomes in terms of microbial assemblages. The structure of each sample is modeled by a hierarchical mixture of multinomial distributions. The model is supplied with labels for one or more factors of interest for each sample and is then trained on a set of samples. This framework enables the model to (*i*) learn how to explain and differentiate factors of interest through its mixture of various assemblages and (*ii*) assign factor values to “future” samples with the appropriate weight. Knights *et al*. [9] applied a Dirichlet prior to a single-level hierarchy and produced a valuable tool for inferring sample provenance; however, that approach necessarily implies near independence among known source environments. Holmes *et al*. [13] extended the Dirichlet prior to a mixture of Dirichlets to facilitate clustering or classification of microbiome samples; in the field of machine learning, this framework is widely known as latent Dirichlet allocation (LDA) [41]. LDA provides a valuable tool for clustering metagenome data [13]; however, it cannot be used to resolve the underlying structure of a community, and the supervised version [13] does not explicitly address the association between community structure and factors of interest.

The hierarchical structure of our model allows “sharing” of potentially compact assemblages across samples, thereby allowing it to capture inter-dependencies between the features of interest and different components of community structure. Additional unique characteristics of our model include the capacity to specify values for any number of known and unknown factor labels and an inference algorithm designed to infer the hyper-parameters of the model from the data. These characteristics permit more accurate inference of the environment-mixture and assemblage-mixture distributions, as well as improve overall model robustness because there is no need to choose and assess values for the hyper-parameters. Although it was not necessary here because the posterior distribution of the hyper-parameters favored small values for the Dirichlet priors, an alternative approach would be to set their values close to zero to induce a “compact” structure for the assemblages and potentially reduce model variance and improve model interpretability [42].

We illustrated the application of our analytical framework to three different serially sampled microbiomes. We used those data to demonstrate that predictive models obtained by using BioMiCo can be used to reliably predict the factor contribution to unlabeled microbiome samples collected at different points in time. Our analysis of human gut and tongue microbiomes revealed that the power to detect temporal stability is improved by using our approach and by exploiting information about microbial assemblages. Previous studies were unable to detect any such signal in those same data. Our analyses of vaginal microbiomes of asymptomatic women identified distinct structural differences between women having normal and elevated Nugent scores, as well as additional assemblage structure within the group having elevated scores. The capacity to detect temporally stable microbial assemblages within human microbiomes suggests future research directions. Longitudinal sampling from both healthy individuals and individuals exhibiting a range of disease intensity could produce models useful for prediction, diagnosis, and treatment of disease. Lastly, our analyses of coastal bacterial communities identified microbial assemblages characteristic of each season, and application of the trained model to a high-resolution dataset validated its accuracy for predicting the pace and timing of seasonal community transitions. These examples illustrate that our approach is effective at learning how community composition is associated with features of interest. Whether working with an ecosystem process (for example, a plankton bloom) or a host phenotype (for example, disease vs. healthy), we anticipate this capability will be especially valuable to understanding and predicting transitions between complex communities composed of hundreds of microbial species.

## Additional files

Additional file 1:
**Analytical solutions for the individual terms of the posterior distribution of the latent variables.** For inference under BioMiCo, we sample from the posterior distribution of latent variables given the data. We use collapsed Gibbs sampling, integrate out the variables *π*, *θ* and *ϕ*, and sample from the posterior distributions of assemblage (*Z*) and factor assignments (*X*). This is a high dimensional distribution, and the analytical solutions for the individual terms are given within this file, along with a general overview of the model. Additional file 2:
**Generating microbial community samples under the model, and validation of the inference algorithm.** This supplement provides an overview of the generative process under BioMiCo, and a detailed description of how we used simulation to validate the inference algorithm. Additional file 3: Figure S1. This figure shows a month-by-month overview of prediction of palm samples (A) and tongue samples (B) from two individuals according to maximum posterior probability. Samples were collected over 7 and 16 months (individuals 1 and 2, respectively) [1]. The white blocks within the plots are the months that were used to train the model. Every row corresponds to the results obtained from a different training month. The height for each row corresponds to the posterior probability scale of 0 to 1. Results for the gut samples from the same two individuals are provided in Figure 2. Additional file 4: Figure S2. This figure identifies the outlier samples that correspond to labeling errors. Fecal samples are shown in panel (A), palm samples are shown in panel (B), and tongue samples are shown in panel (C). The plots show the prediction of both human host identity and body site according to maximum posterior probability, with the outliers indicated by arrows and labeled with sample IDs. The original analysis by Caporaso *et al*. [1] excluded these outliers. Samples were from the gut, palm, and tongue of two individuals collected over 7 and 16 months (individuals 1 and 2, respectively) [1]. The white blocks within the plots are the months that were used to train the model. Every row corresponds to the results obtained from a different training month. The height for each row corresponds to the posterior probability scale of 0 to 1. Results for the gut samples from the same two individuals are provided in Figure 2.

## Abbreviations

AE: autumn equinox
BioMiCo: Bayesian inference of microbial communities
BV: bacterial vaginosis
KEGG: Kyoto Encyclopedia of Genes and Genomes
LDA: latent Dirichlet allocation
OTU: operational taxonomic unit
PD: posterior density
SE: spring equinox
SS: summer solstice
WGS: whole genome sequencing
WS: winter solstice
