# Supplementary material for: BioMiCo: a supervised Bayesian model for inference of microbial community structure
Source: Microbiome. 2015 Mar 10;3:8. doi: 10.1186/s40168-015-0073-x (PMC4359585; doi:10.1186/s40168-015-0073-x)
Supplement: Additional file 1: — Analytical solutions for the individual terms of the posterior distribution of the latent variables. For inference under BioMiCo, we sample from the posterior distribution of latent variables given the data. We use collapsed Gibbs sampling, integrate out the variables π, θ and ϕ, and sample from the posterior distributions of assemblage (Z) and factor assignments (X). This is a high dimensional distribution, and the analytical solutions for the individual terms are given within this file, along with a general overview of the model. [file 40168_2015_73_MOESM1_ESM.pdf]

ADDITIONAL FILE 1: Model overview with analytical solution for the posterior distribution of latent variables

We assume that each microbiome sample is a mixture of OTUs from one or more communities. We model the community mixture by using K pre-specified factors as mixture components, where factors serve as proxies for putative communities and their relative importance is permitted to differ between samples. The relative contribution of each factor to the nth sample is modeled through the latent variable π_n. If a sample can be assumed, *a priori,* to reflect the contribution of as many as, say, 4 factors, then π_n is a probability vector of 4 non-zero values summing to one. Because we only require that some subset of factors contribute to a given sample, the probabilities of other non-contributing factors are set to zero.

Each putative community that is represented by a pre-specified factor is comprised of a fixed number of OTU assemblages (L). The contribution of each assemblage to the kth factor, θ_k , is modeled by a vector of L mixing probabilities that sum to one. The element θ_{kl} in a $K \times L$ matrix θ represents the relative contribution of assemblage l to factor k.

Each assemblage is comprised of a mixture of T different OTUs. The contribution of each OTU to the lth assemblage, ϕ_l , is modeled by a vector of T mixing probabilities that sum to one. The element ϕ_{li} in $L \times T$ matrix ϕ represents the relative contribution of OTU i in assemblage l.

We assume an independent and identical (iid) symmetric Dirichlet priors on π_n and on the rows of θ and ϕ:

$$\pi_n \sim Dirichlet(\alpha_\pi)$$
$$\theta_k \sim Dirichlet(\alpha_\theta)$$
$$\phi_l \sim Dirichlet(\alpha_\phi)$$

The complete likelihood of the data given the hyper-parameters of the prior distributions is

$$P\big(X, Z, W, \pi, \theta, \phi \big| \alpha_\pi, \alpha_\theta, \alpha_\phi\big) =$$

$$P(X|\pi)P(Z|X,\theta)P(W|Z,\phi)\ P(\pi|\alpha_\pi)\ P(\theta|\alpha_\theta)P\big(\phi\big|\alpha_\phi\big) =$$

$$\prod_{n=1}^{N}\prod_{i=1}^{N_n} P(X_{ni}\,|\pi_n) \times \prod_{n=1}^{N}\prod_{i=1}^{N_n} P(Z_{ni}\,|\theta_{X_{ni}}) \times \prod_{n=1}^{N}\prod_{i=1}^{N_n} P(W_{ni}\,|\phi_{Z_{ni}}) \times$$

$$P(\pi|\alpha_\pi)P(\theta|\alpha_\theta)P(\phi|\alpha_\phi)$$

where N is the number of samples in a dataset and N_n is the number of OTUs in sample n. Z and X represent the assemblage and factor assignments for each OTU in all samples, and X_{ni} and Z_{ni} represent the factor and assemblage assignments for OTU i in sample n. Note that Z_{ni} is a value between 1 and L (number of assemblages) and X_{ni} is a value between 1 and K (number of factors). W_{ni} is the OTU i in sample n, and has a value between 1 and T.

For posterior inference, we need to sample from the posterior distribution of latent variables given the data:

$$P(X, Z, \pi, \theta, \phi | W, \alpha_\pi, \alpha_{\theta,} \alpha_\phi)$$

where Z, Y, π, θ and ϕ are the latent variables in the model. We use collapsed Gibbs sampling, integrate out the variables π, θ and ϕ, and sample from the posterior distributions of assemblage (Z) and factor assignments (X). Employing Gibbs sampling, we sample the assemblage and factor assignments for OTU i in sample n (Z_{ni}, X_{ni}) given all other assemblage and factor assignments for OTUs in all samples excluding the current OTU. Sampling of the assemblage and factor assignments is based on the following conditional probability:

$$P(Z_{ni}, X_{ni} | W, Z_{-ni}, X_{-ni}) =$$

$$\frac{\iiint P(X, Z, W, \pi, \theta, \phi)\ d\pi\ d\theta\ d\phi}{\iiint P(X_{-ni}, Z_{-ni}, W, \pi, \theta, \phi)\ d\pi\ d\theta\ d\phi} =$$

$$\frac{\int P(X|\pi)P(\pi|\alpha_\pi)\ d\pi}{\int P(X_{-ni}|\pi)P(\pi|\alpha_\pi)\ d\pi} \times \frac{\int P(Z|X,\theta)P(\theta|\alpha_\theta)\ d\theta}{\int P(Z_{-ni}|X_{-ni},\theta)P(\theta|\alpha_\theta)\ d\theta} \times \frac{\int P(W|Z,\phi)P\big(\phi\big|\alpha_\phi\big)\ d\phi}{\int P(W|Z_{-ni},\phi)P\big(\phi\big|\alpha_\phi\big)\ d\phi}$$

Solutions to integrals in the above equation can be analytically derived:

$$\int P(X|\pi)P(\pi|\alpha_\pi)\,d\pi =$$

$$\int \prod_{n=1}^{N}\prod_{i=1}^{N_n} P(X_{ni}|\pi_n)\prod_{n=1}^{N} P(\pi_n|\alpha_\pi)\,d\pi =$$

$$\int \prod_{n=1}^{N}\prod_{k=1}^{K} \pi_{nk}^{C_n^k}\prod_{n=1}^{N}\frac{\Gamma(K\alpha_\pi)}{\Gamma(\alpha_\pi)^K}\prod_{k=1}^{K}\pi_{nk}^{\alpha_\pi-1}\,d\pi =$$

$$\prod_{n=1}^{N}\frac{\Gamma(K\alpha_\pi)}{\Gamma(\alpha_\pi)^K}\frac{\prod_{k=1}^{K}\Gamma(\alpha_\pi + C_n^k)}{\Gamma\left(\sum_{k=1}^{K}\left(\alpha_\pi + C_n^k\right)\right)}$$

where C_n^k the number of times that an OTU in the nth sample is assigned to the kth factor.

$$\int P(Z|X,\theta)P(\theta|\alpha_\theta)\,d\theta =$$

$$\int \prod_{n=1}^{N}\prod_{i=1}^{N_n} P(Z_{ni}|X_{ni},\theta)\prod_{k=1}^{K} P(\theta_k|\alpha_\theta)\,d\theta =$$

$$\int \prod_{k=1}^{K}\prod_{l=1}^{L} \theta_{kl}^{C_k^l}\prod_{k=1}^{K}\frac{\Gamma(L\alpha_\theta)}{\Gamma(\alpha_\theta)^L}\prod_{l=1}^{L}\theta_{kl}^{\alpha_\theta-1}\,d\theta =$$

$$\prod_{k=1}^{K}\frac{\Gamma(L\alpha_\theta)}{\Gamma(\alpha_\theta)^L}\frac{\prod_{l=1}^{L}\Gamma\left(\alpha_\theta + C_k^l\right)}{\Gamma\left(\sum_{l=1}^{L}\left(\alpha_\theta + C_k^l\right)\right)}$$

where C_k^l is the number of times that an OTU in the kth factor is assigned to the lth assemblage.

$$\int P(W|Z,\phi)P(\phi|\alpha_\phi)\,d\phi =$$

$$\int \prod_{n=1}^{N}\prod_{i=1}^{N_n} P(W_{ni}|Z_{ni},\theta)\prod_{l=1}^{L} P(\phi_l|\alpha_\phi)\,d\phi =$$

$$\int \prod_{l=1}^{L}\prod_{t=1}^{T} \phi_{lt}^{C_t^l}\prod_{l=1}^{L}\frac{\Gamma(T\alpha_\phi)}{\Gamma(\alpha_\phi)^T}\prod_{t=1}^{T}\phi_{lt}^{\alpha_\phi-1}\,d\phi =$$

$$\prod_{l=1}^{L}\frac{\Gamma(T\alpha_\phi)}{\Gamma(\alpha_\phi)^T}\frac{\prod_{t=1}^{T}\Gamma(\alpha_\phi + C_t^l)}{\Gamma(\sum_{t=1}^{T}(\alpha_\phi + C_t^l))}$$

where C_t^l is the number of times that OUT t in is assigned to the lth assemblage, with the value of t between 1 and T.

Given the above conditional probability we use Gibbs sampling for drawing samples from the posterior distribution $P(X_{ni} = k, Z_{ni} = l | X_{-ni}, Z_{-ni}, W, \alpha_\pi, \alpha_\theta, \alpha_\phi)$ where Z_{-ni} represents the assemblage assignment for all OTUs in all samples except only the ith OTU in n^{th} microbiome sample, and X_{-ni} represents the factor assignment for all OTUs in all samples except only the ith OTU in n^{th} microbiome sample**.** At each iteration of Gibbs sampling, we repeat the following in a random order. For each OTU in each sample, we draw the assemblage and factor assignment (Z_{ni} and X_{ni}) of an OTU given the current assemblage and factor assignments of all other OTUs in this and every other sample. From the following equation, a value is calculated for each possible combination of values for X_{ni} and Z_{ni}.

$$P(X_{ni} = k, Z_{ni} = l | X_{-ni}, Z_{-ni}, W, \alpha_\pi, \alpha_\theta, \alpha_\phi) =$$

$$\frac{\alpha_\pi + C_n^k}{\sum_{k'}(\alpha_\pi + C_n^{k'})}\times\frac{\alpha_\theta + C_k^l}{\sum_{l'}(\alpha_\theta + C_k^{l'})}\times\frac{\alpha_\phi + C_{W_{ni}}^l}{\sum_{w'}(\alpha_\phi + C_{W'_{ni}}^l)}$$

Note that X_{ni} can take only factor assigments that are known to be contributing to sample *n*. These values are then normalized and used to draw new assignment values for X_{ni} and Z_{ni} which are immediately updated for use in the next iteration. The hyper-parameters of our model (α_π, α_θ, and α_ϕ) can be fixed by the user, or MCMC sampling can be used to learn them from the training

data. In the latter case we can employ Metropolis-within-Gibbs sampling scheme where Metropolis-Hastings updates are used to resample new values for hyper-parameters once all the other parameters of the model have been sampled.
