# Supplementary material for: BioMiCo: a supervised Bayesian model for inference of microbial community structure
Source: Microbiome. 2015 Mar 10;3:8. doi: 10.1186/s40168-015-0073-x (PMC4359585; doi:10.1186/s40168-015-0073-x)
Supplement: Additional file 2: — Generating microbial community samples under the model, and validation of the inference algorithm. This supplement provides an overview of the generative process under BioMiCo, and a detailed description of how we used simulation to validate the inference algorithm. [file 40168_2015_73_MOESM2_ESM.pdf]

ADDITIONAL FILE 2: Simulation and validation of the inference algorithm

Simulation:

Our model is a generative model; *i.e.*, it assumes a process by which data is generated. Therefore, it is straightforward to simulate data for the purpose of testing the inference algorithm. For each of i OTUs in microbiome sample n:

- Environmental factor $X_{ni} \in \{1,2,\cdots,K\}$ is drawn from the factor mixture π_n associated with sample n.
- Assemblage $Y_{ni} \in \{1,2,\cdots,L\}$ is drawn from the mixture of assemblages associated with factor Z_{ni} that was drawn in the previous step. The assemblage is chosen by sampling from the row Z_{ni} of matrix θ (*i.e.*, $\theta_{Z_{ni}}$).
- Choose the OTU for assemblage Y_{ni} by sampling from row Y_{ni} of matrix ϕ (*i.e.*, $\phi_{Y_{ni}}$).

This generative process is repeated for every OTU in a simulated microbiome sample n. The same process generates the OTU composition of all microbiome samples.

We simulated and analyzed data to (*i*) verify that our sampling algorithm can recover the parameter values used to generate structured microbiome samples in the training phase, and (*ii*) to investigate what conditions represent easy and hard classification problems for the testing phase. Hence, our simulation design is based on covering a very wide range of scenarios. We simulated different

scenarios by selecting the number of environmental factors, assemblages and OTUs from (K = 4, 8, 12), (L = 5, 10, 30, 50, 70) and (T = 500, 1000, 2000, 4000) respectively. Analyses of these simulated data are computationally costly. Because it is unlikely that there will be highly complex assemblage structure in real data having low numbers of OTUs, to save on computational costs we did not investigate very large numbers of assemblages (50 and 70) for communities having low numbers of OTUs (e.g., 500). The number of assemblages we investigated relative to the number of OTUs are given in Table 1 below. Note that for the largest number of OTUs (4000) we did investigate the full rage of assemblage sizes (5 to 70). Lastly, because we do not expect unknown microbiome samples to be identical in their assemblage composition we generated test samples according to a variety of mixtures (Appendix 1).

Table 1. OTU counts vary according to the number of assemblages in the generating model.

	Assemblages				
**OTUs**	5	10	30	50	70
500	+	+	-	-	-
1000	+	+	+	-	-
2000	+	+	+	+	-
4000	+	+	+	+	+

Simulated communities had numbers of OTUs ≥30× the number of assemblages. A plus sign (+) indicates where assemblage counts satisfied this condition, and hence were evaluated as part of the simulated study. A minus sign (-) indicates a combination of OTU and assemblage count that was not evaluated.

Given the above range of scenarios, we also generated under a wide range of community structures. In our simulation the concentration parameter for the Dirichlet prior, α_ϕ, controls the amount of mixing among OTUs contributing to assemblages. We employed values of 0.01, 0.05 and 0.25. The higher the

concentration parameter, the more evenly distributed the OTUs will be among assemblages (*i.e.*, less signal), and consequently the more the difficult this type of structure is for the testing phase of the inference algorithm. As can be seen in Figure 1, α_ϕ = 0.25 yields a more even distribution within the unit simplex (*i.e.*, the distribution is less concentrated in the corners as compared to when α_ϕ is small). As α_ϕ = 0.25 represents a much less structured community, it poses a more significant analytical challenge.

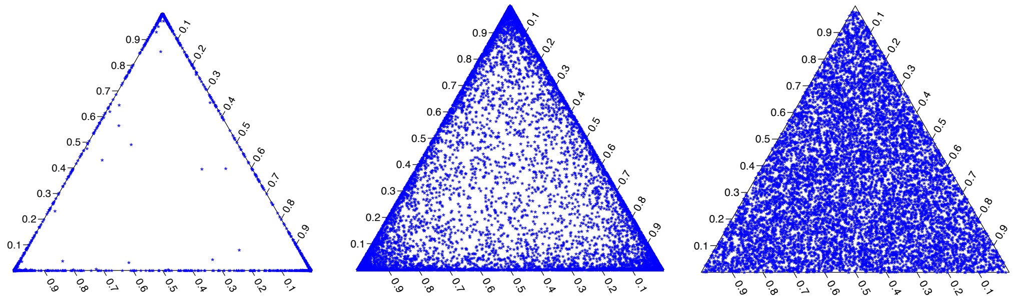

Figure 1. Samples (10,000 independent samples) drawn from Dirichlet distributions with concentration parameters equal to 0.01 (left), 0.25 (centre) and 1.0 (right). Note that dimension is K=3 here for illustrative purposes; simulations were carried out by using K = 4, 8, 12. Also note how a significant number of samples drawn from a Dirichlet distribution with concentration parameter of 0.25 (centre) have mixed membership to all three groups, and how the dsitribution is much more evenly distributed as compared to a concentration parameter of 0.01. A concentration parameters equal to 1.0 (right) yields uniform samples within the simplex.

The concentration parameter α_θ controls the amount of mixing among assemblages contributing to factors. The higher the concentration parameter, the more evenly distributed the assemblages will be among the various environmental factors. Again, we employed values of 0.01, 0.05 and 0.25.

We added an extreme case by setting α_θ= 1.0. This completely removes from the testing phase the added information-value of having assemblage structure within the data (see Figure 1 for an ecample). Such data represent a highly challenging classification task when combined with α_ϕ = 0.25, as the only signal in the data resides in OTUs that will be widely shared among environmental factors. Thus we have three scenarios that constitute a convenient reference for training and testing: (*i*) "easy" datasets, where α_ϕ = 0.01 and α_θ= 0.01, (*ii*) "hard" datasets, where α_ϕ = 0.25 and α_θ= 0.25, and (*iii*) "extreme" datasets, where α_ϕ = 0.25 and α_θ= 1.0. The remaining scenarios we evaluated represent a wide range of conditions between "easy" and "hard". Taken together, we evaluated a total of 504 different scenarios.

Assessment of the inference algorithm:

We validated the inference algorithm by comparing the values of each hyper-parameter specified in the generating model (α_ϕ and α_θ) to the values estimated by using Metropolis-Hastings during the training phase. We obtained reasonable estimates in the "easy", "hard" and even the "extreme" scenarios (Table 2), indicating the model did infer hierarchical structure when present, as well as indicate when it is absent (as was the case in the "extreme" scenario, where the inferred values of α_θ were close 1.0). Note that any inference from a finite number of MCMC samples can only approximate the target distribution, and the number of steps required for convergence with a similar amount of error will differ among

datasets. Given the computational cost of a large-scale simulation study, and that all the simulated datasets were run to the same length (5000 iterations; 500 burn-in with sampling every 250 thereafter), some differences among scenarios in the approximation of the target distribution (Table 2) are expected.

Table 2. Inferred values for the hyper-parameters of BioMCo

	**"easy"**		**"hard"**		**"extreme"**	
	α_ϕ=0.01	α_θ=0.01	α_ϕ=0.25	α_θ=0.25	α_ϕ=0.25	α_θ=1.0
***K*=4**						
L=5	0.010	0.031	0.246	0.393	0.241	0.815
L=10	0.010	0.017	0.252	0.298	0.229	0.940
L=30	0.010	0.018	0.287	0.214	0.239	1.038
L=50	0.011	0.012	0.227	0.332	0.231	1.058
***K*=8**						
L=5	0.010	0.023	0.240	0.385	0.243	1.004
L=10	0.011	0.011	0.244	0.310	0.244	0.966
L=30	0.010	0.012	0.257	0.216	0.230	1.128
L=50	0.011	0.011	0.267	0.212	0.234	1.100
***K*=12**						
L=5	0.010	0.015	0.241	0.276	0.233	1.141
L=10	0.011	0.013	0.246	0.277	0.238	1.028
L=30	0.010	0.013	0.253	0.249	0.243	1.027
L=50	0.010	0.010	0.249	0.280	0.250	0.877

The hyper-parameters of BioMiCo are the concentration parameters of two symmetric Dirichlet distributions.

Given that hierarchal structure was detected in the "easy" and "hard" scenarios, we also assessed the inference of assemblage mixing probabilities in selected scenarios. When there is hierarchical signal, the model-derived assemblages could be easily coordinated with the structure of the generating model by eye when there are not too many assemblages to inspect (*i.e.*, when L = 5 or 10). We did this for both "easy" and "hard" datasets for K=4, 8 and 12 environmental factors, and compared the inferred assemblage mixing probabilities to those used

to generate the data by using the Jensen–Shannon divergence (JSD: [1]). The low JSD scores presented in Table 3 illustrate that the assemblage mixing probabilities were reliably estimated.

Table 3. Similarity between the inferred and generating distribution of assemblages, as measured by the Jensen–Shannon divergence.

**"Easy" (α_ϕ = 0.01 and α_θ= 0.01)**		
	**Number of assemblages**	
**Factors**	***L*** **= 5**	***L*** **= 10**
K **= 4**	0.00021	0.00021
K **= 8**	0.00002	0.04079
K **= 12**	0.00001	0.00006
**"Hard" (α_ϕ = 0.25 and α_θ= 0.25)**		
	**Number of assemblages**	
**Factor**	***L*** **= 5**	***L*** **= 10**
K **= 4**	0.00105	0.02374
K **= 8**	0.00215	0.00489
K **= 12**	0.00082	0.00410

The Jensen–Shannon divergence (JSD) quantifies the similarity between two probability distributions. JSD=0 when the two distributions are identical. JSD=1 at its maximum (when the two distributions are distinct).

Given that we obtained reliable inferences within the training phase, we next investigated all the scenarios between "easy" and "hard" so as to investigate the inference of mixing probabilities for the environmental labels in the test dataset. It is with respect to these values that real datasets will be classified according to environmental factors. Figure 2 presents a heat map of the mean JSD scores (over 103 test samples per scenario) between the true mixing probabilities for each test sample, and those inferred by BioMiCo. Blue colors indicate very good estimation of the mixture weights for each environmental factor. White indicates

some divergence, and red indicates highly divergent inferences. In no case did we obtain highly divergent estimates. The dominance of low divergences (blue) in Figure 2 indicates that the inference algorithm is capable of learning which assemblages are associated with different environmental variables, and reliably inferring the mixture weights for environmental variables, even when unlabeled test samples are unique mixtures of assemblages that were not encountered in the training phase.

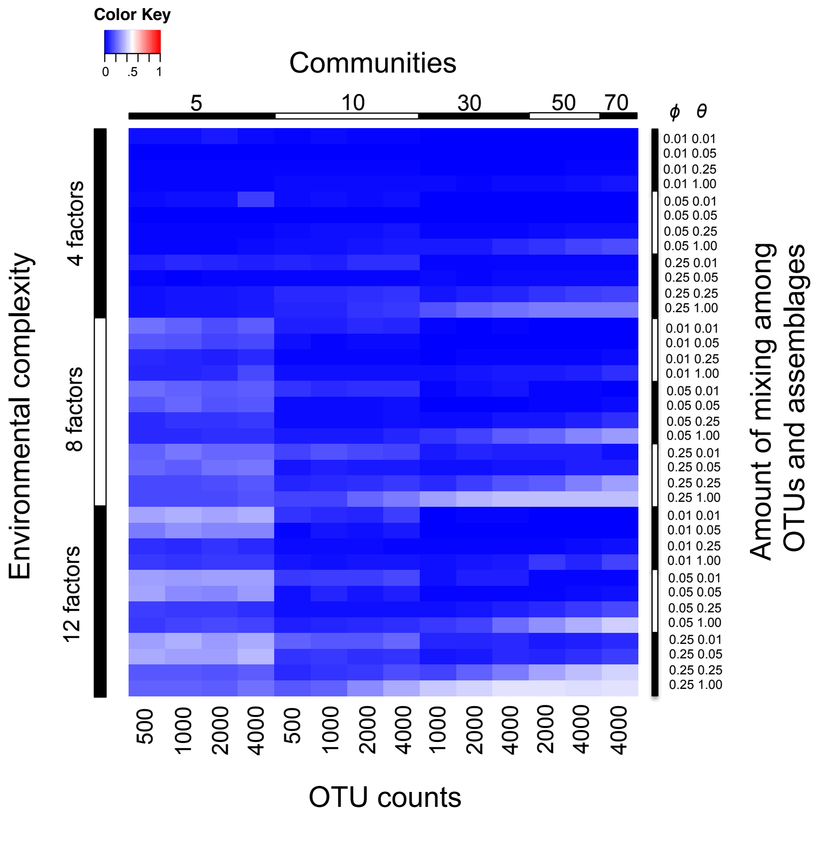

Figure 2. Heatmap of mean Jensen Shannon divergence scores between the true mixing probabilities for each test set of data, and those inferred by BioMiCo. This plot covers 504 different scenarios, and for each one there was a test set of data comprised of 103 microbiome samples. The values shown in each cell of this heatmap are the average of 103 JSD scores per scenario. The number of distinct scenarios is due to differences in OTU counts (500, 1000, 2000, 4000), number of communities (5, 10, 30, 50, 70), environmental complexity (4, 8, 12 factors) and mixing values among OTUs (θ) (0.01, 0.05, 0.25) and assemblages (ϕ) (0.01, 0.05, 0.25 and 1.0).

The results in Figure 2 do reveal the conditions that pose the biggest challenges to inference (indicated by light blue to white). The most challenging conditions reflect two type of scenarios: (*i*) few assemblages (L=5) with many factors (K=8 and 12), and (*ii*) communities with very little structure ("hard" and "extreme" cases: α_{ϕ} = 0.25 and α_{θ}= 0.25 or 1.0) divided among a large numbers of assemblages (L=30, 50 and 70). In the first type of scenario, classification was attempted according to 8 and 12 factor labels. In these cases the community structure was concentrated in a very small number of assemblages, and the training samples had low mixture complexity; this yielded too little signal to support classification of individual samples according to so many different environmental factors. We expect that this will be the case whenever K is greater than L. Note that classification was highly reliable when there were just 4 factor labels, or more complex community structures (Figure 2). In the second type of scenario, a limited amount of community structure was spread across a very large number of assemblages, thereby diluting the available signal. These two types of conditions represent two different ways in which the boundary on performance might ultimately be reached in real data: (*i*) attempting classification for too many factor labels in structured, but low complexity, data and (*ii*) attempting even simple classification from very weakly structured data.

Users of BioMiCo should note that performance also can be assessed for their own real datasets as long as they withhold a portion of their labeled samples for the purpose of cross validating their trained-model. If the training data is too small to withhold a large number of labeled samples, then they can use a technique

called “leave-one-out cross validation” that aggregates the predictive accuracy for each sample in the training data by “hiding” each sample, in turn, from the model and testing the model’s predictions for that sample [2]. At the end of a complete “leave-one-out rotation”, the performance of the model for real data can then be measured as the percent of samples that were correctly classified [3].

References:

1. Lin, J. (1991) Divergence measures based on the shannon entropy. *IEEE Transactions on Information Theory* 37(1):145–151.
2. Hastie, T., Tibshirani, R., Friedman, J., Hastie, T., Friedman, J., and Tibshirani, R. (2009) The elements of statistical learning, 2nd edn. USA: Springer.
3. El-Swais, H., Dunn, K.A., Bielawski, J.P., Li, W.W.K., Walsh, D.A. (2014) Seasonal assemblages and short-lived blooms in northwest Atlantic Ocean bacterioplankton. *Environmental Microbiology*. (In Press).

APPENDIX: Environment labels for test and training data samples.

K=4				*K*=8				*K*=12			
**Training**		**Test**		**Train**		**Test**		**Train**		**Test**	
**ID**	**Label**	**ID**	**Label**	**ID**	**Label**	**ID**	**Label**	**ID**	**Label**	**ID**	**Label**
2	E2	1	E1	2	E3	1	E1	2	E4	1	E2
4	E1	3	E1	4	E1	3	E1	4	E1	3	E1
5	E4	6	E2	5	E6	6	E3	5	E9	6	E5
7	E4	10	E2,E1,E4	7	E6	10	E3,E2,E5	7	E8	10	E4,E2,E8
8	E3	12	E2	8	E5	12	E4	8	E6	12	E6
9	E1	15	E1,E2	9	E1	15	E1,E4	9	E12	15	E2,E6
11	E3	17	E3,E1,E2,E4	11	E4	17	E5,E2,E4,E8	11	E6	17	E7,E2,E12,E9
13	E3	18	E2,E4,E3	13	E4	18	E4,E8,E6	13	E5	18	E6,E12,E10
14	E2	19	E1,E4,E3	14	E3	19	E2,E8,E1	14	E4	19	E3,E4,E1
16	E2	29	E2	16	E3	29	E3	16	E4	29	E5
20	E3	30	E2,E1	20	E5	30	E3,E2	20	E7	30	E4,E3
21	E3	35	E3,E1,E4	21	E4	35	E6,E3,E7	21	E5	35	E8,E4,E11
22	E2	36	E1,E4	22	E3	36	E1,E2	22	E4	36	E2,E12
23	E4	38	E1,E2,E3	23	E7	38	E2,E3,E5	23	E10	38	E2,E4,E7
24	E2	39	E4	24	E2	39	E8	24	E2	39	E12
25	E4	40	E3	25	E7	40	E6	25	E10	40	E9
26	E3	41	E3,E1	26	E4	41	E6,E1	26	E6	41	E8,E1
27	E3	42	E1	27	E5	42	E2	27	E6	42	E2
28	E1	43	E2	28	E8	43	E4	28	E11	43	E6
31	E1	44	E3	31	E1	44	E6	31	E1	44	E9
32	E3	45	E1	32	E4	45	E2	32	E5	45	E3
33	E1	46	E1,E3	33	E1	46	E1,E7	33	E1	46	E1,E10
34	E4	47	E3	34	E7	47	E5	34	E10	47	E7
37	E4	48	E4,E1,E2	37	E7	48	E8,E1,E6	37	E10	48	E12,E1,E9
50	E2	49	E3,E1	50	E2	49	E5,E1	50	E2	49	E7,E1
53	E3	51	E4,E2	53	E5	51	E7,E4	53	E6	51	E10,E7
55	E3	52	E1,E4,E2	55	E5	52	E2,E8,E5	55	E7	52	E2,E4,E7
58	E4	54	E4	58	E6	54	E8	58	E8	54	E12
59	E4	56	E1,E3,E2,E4	59	E7	56	E1,E7,E6,E4	59	E10	56	E1,E10,E9,E7
61	E1	57	E4	61	E1	57	E8	61	E12	57	E12
65	E4	60	E2,E1,E4	65	E6	60	E4,E1,E8	65	E9	60	E5,E1,E7
67	E3	62	E4,E1,E2	67	E5	62	E8,E1,E4	67	E7	62	E11,E1,E6
68	E4	63	E1,E2,E4	68	E6	63	E2,E4,E3	68	E8	63	E3,E6,E5
69	E2	64	E2,E1	69	E2	64	E3,E1	69	E2	64	E4,E1
71	E3	66	E4	71	E4	66	E7	71	E5	66	E11
72	E3	70	E3,E2	72	E5	70	E5,E3	72	E6	70	E7,E5
73	E2	74	E2,E1	73	E3	74	E4,E1	73	E4	74	E5,E1
78	E1	75	E4,E2,E3	78	E8	75	E7,E5,E8	78	E11	75	E11,E7,E8
82	E2	76	E1	82	E3	76	E1	82	E3	76	E1
84	E1	77	E3	84	E8	77	E6	84	E11	77	E9
87	E4	79	E3	87	E6	79	E5	87	E8	79	E7
88	E4	80	E3,E4	88	E7	80	E6,E7	88	E9	80	E8,E11
89	E2	81	E4,E1	89	E3	81	E7,E2	89	E4	81	E10,E3
92	E4	83	E1,E2	92	E7	83	E2,E4	92	E9	83	E3,E6
94	E3	85	E4	94	E4	85	E8	94	E5	85	E12
97	E4	86	E3	97	E7	86	E6	97	E10	86	E9
100	E3	90	E1	100	E5	90	E2	100	E6	90	E2
101	E1	91	E4,E3	101	E1	91	E8,E6	101	E1	91	E12,E9
104	E1	93	E1	104	E1	93	E1	104	E1	93	E2
106	E4	95	E1,E4,E3	106	E7	95	E1,E2,E8	106	E10	95	E1,E3,E2
107	E3	96	E4	107	E4	96	E8	107	E5	96	E11
108	E2	98	E2,E1	108	E3	98	E3,E2	108	E4	98	E4,E2
111	E4	99	E3,E1,E2	111	E6	99	E6,E1,E5	111	E9	99	E9,E1,E7
114	E3	102	E3	114	E4	102	E5	114	E5	102	E7
115	E4	103	E1,E2,E4	115	E6	103	E1,E3,E8	115	E8	103	E1,E5,E12
117	E1	105	E4,E1,E3	117	E8	105	E7,E1,E8	117	E11	105	E10,E1,E11
118	E2	109	E4	118	E3	109	E7	118	E4	109	E10
119	E3	110	E2,E4	119	E5	110	E3,E4	119	E6	110	E5,E7
121	E3	112	E1,E2,E3,E4	121	E5	112	E2,E4,E6,E5	121	E7	112	E2,E6,E9,E8
126	E1	113	E4,E2	126	E8	113	E8,E5	126	E12	113	E12,E8
130	E1	116	E3,E2,E1	130	E1	116	E5,E4,E1	130	E1	116	E7,E12,E2
131	E1	120	E2	131	E1	120	E3	131	E12	120	E5
132	E4	122	E4,E3	132	E7	122	E8,E6	132	E10	122	E11,E9
133	E1	123	E4,E2	133	E1	123	E7,E5	133	E1	123	E10,E8
134	E4	124	E4,E1	134	E6	124	E7,E3	134	E8	124	E10,E4

135	E4	125	E3,E2,E1	135	E6	125	E5,E8,E3	135	E8	125	E7,E8,E5
136	E3	127	E3	136	E4	127	E5	136	E6	127	E7
137	E3	128	E1,E2	137	E4	128	E2,E4	137	E6	128	E2,E7
138	E2	129	E2	138	E2	129	E4	138	E2	129	E5
139	E4	140	E3	139	E6	140	E6	139	E8	140	E9
145	E1	141	E3,E1	145	E8	141	E5,E1	145	E12	141	E7,E2
150	E2	142	E1	150	E2	142	E1	150	E2	142	E1
151	E2	143	E4,E1	151	E2	143	E7,E2	151	E2	143	E10,E3
157	E2	144	E1	157	E3	144	E2	157	E3	144	E2
161	E1	146	E1	161	E8	146	E2	161	E11	146	E3
163	E4	147	E3,E4	163	E7	147	E5,E7	163	E10	147	E8,E11
166	E1	148	E3	166	E1	148	E5	166	E1	148	E7
167	E3	149	E1	167	E5	149	E1	167	E7	149	E1
171	E2	152	E3,E4	171	E2	152	E5,E6	171	E2	152	E7,E8
173	E4	153	E4	173	E7	153	E8	173	E9	153	E12
174	E1	154	E1	174	E8	154	E2	174	E11	154	E2
175	E2	155	E4	175	E3	155	E7	175	E3	155	E11
176	E3	156	E1	176	E5	156	E1	176	E6	156	E2
178	E2	158	E2,E4	178	E3	158	E4,E5	178	E4	158	E5,E8
179	E2	159	E3,E2,E1	179	E2	159	E6,E5,E1	179	E2	159	E9,E7,E2
180	E3	160	E4,E2,E3	180	E5	160	E8,E4,E5	180	E6	160	E11,E6,E8
181	E2	162	E2,E3,E1	181	E2	162	E4,E6,E2	181	E2	162	E5,E9,E2
183	E2	164	E4,E1	183	E3	164	E8,E3	183	E4	164	E12,E4
185	E2	165	E1,E4	185	E2	165	E1,E2	185	E2	165	E1,E3
188	E4	168	E4,E2	188	E7	168	E7,E4	188	E10	168	E11,E7
189	E3	169	E3,E1,E2	189	E4	169	E5,E2,E8	189	E5	169	E7,E4,E8
190	E2	170	E4	190	E2	170	E7	190	E3	170	E10
193	E2	172	E3,E4	193	E2	172	E5,E6	193	E2	172	E7,E10
194	E1	177	E2,E1	194	E1	177	E4,E1	194	E12	177	E6,E1
195	E1	182	E4,E2,E1	195	E8	182	E7,E4,E2	195	E11	182	E11,E6,E4
198	E1	184	E1	198	E8	184	E2	198	E11	184	E2
200	E1	186	E2	200	E1	186	E4	200	E1	186	E6
		187	E2			187	E3			187	E4
		191	E2			191	E4			191	E5
		192	E1,E2,E4,E3			192	E1,E5,E3,E2			192	E1,E7,E5,E4
		196	E3,E4,E1			196	E5,E7,E1			196	E7,E11,E1
		197	E4,E2			197	E8,E4			197	E12,E6
		199	E4,E2,E1			199	E7,E3,E8			199	E10,E5,E4
