# Supplementary material for: BioMiCo: a supervised Bayesian model for inference of microbial community structure
Source: Microbiome. 2015 Mar 10;3:8. doi: 10.1186/s40168-015-0073-x (PMC4359585; doi:10.1186/s40168-015-0073-x)
Supplement: Additional file 3: Figure S1. — This figure shows a month-by-month overview of prediction of palm samples (A) and tongue samples (B) from two individuals according to maximum posterior probability. Samples were collected over 7 and 16 months (individuals 1 and 2, respectively) [1]. The white blocks within the plots are the months that were used to train the model. Every row corresponds to the results obtained from a different training month. The height for each row corresponds to the posterior probability scale of 0 to 1. Results for the gut samples from the same two individuals are provided in Figure 2. [file 40168_2015_73_MOESM3_ESM.pdf]

Figure S1A

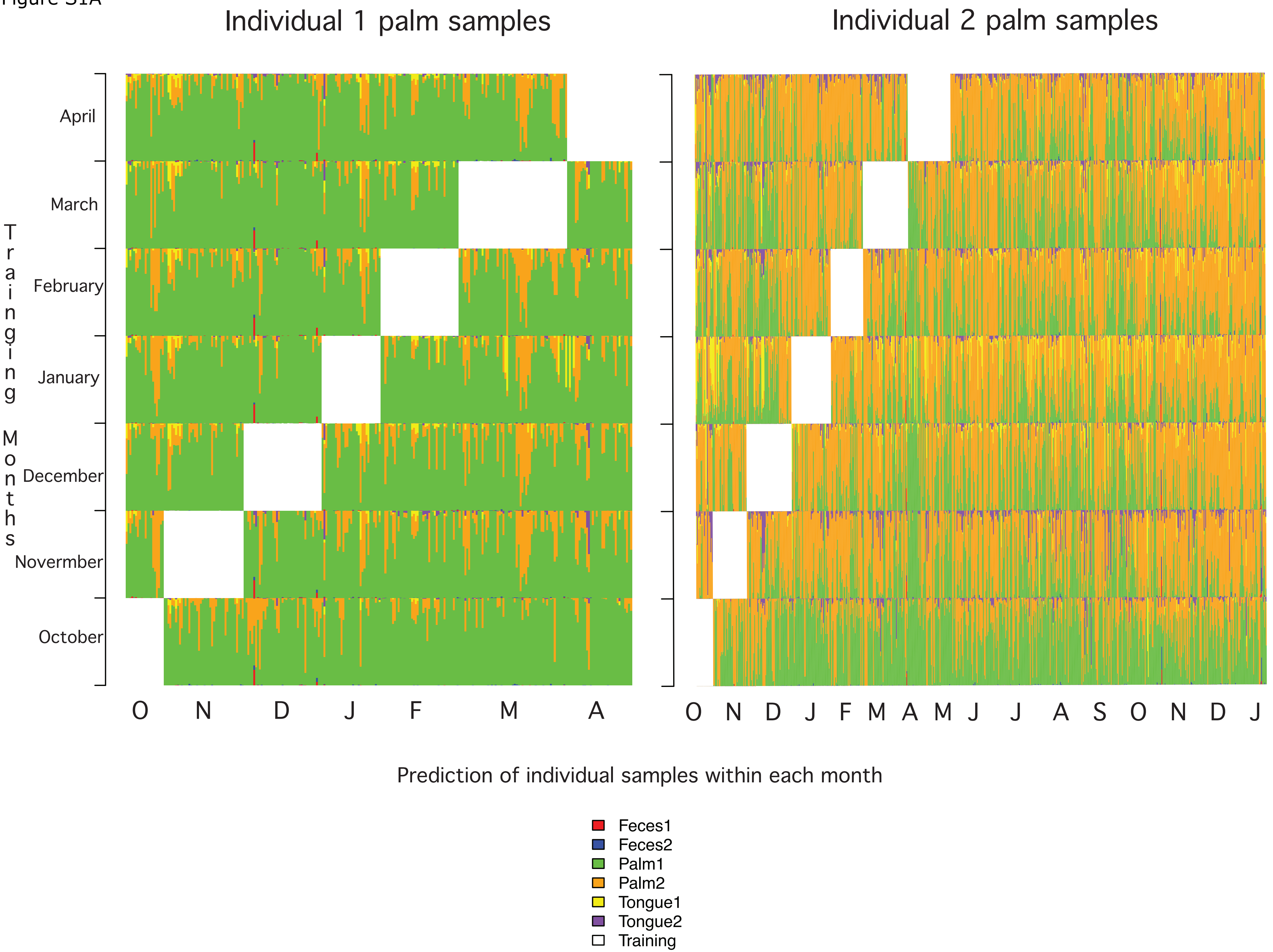

Individual 1 palm samples
Individual 2 palm samples
Training Months
April
March
February
January
December
Novermber
October
O N D J F M A
O N D J F M A M J J A S O N D J
Prediction of individual samples within each month
Feces1
Feces2
Palm1
Palm2
Tongue1
Tongue2
Training

Figure S1 B

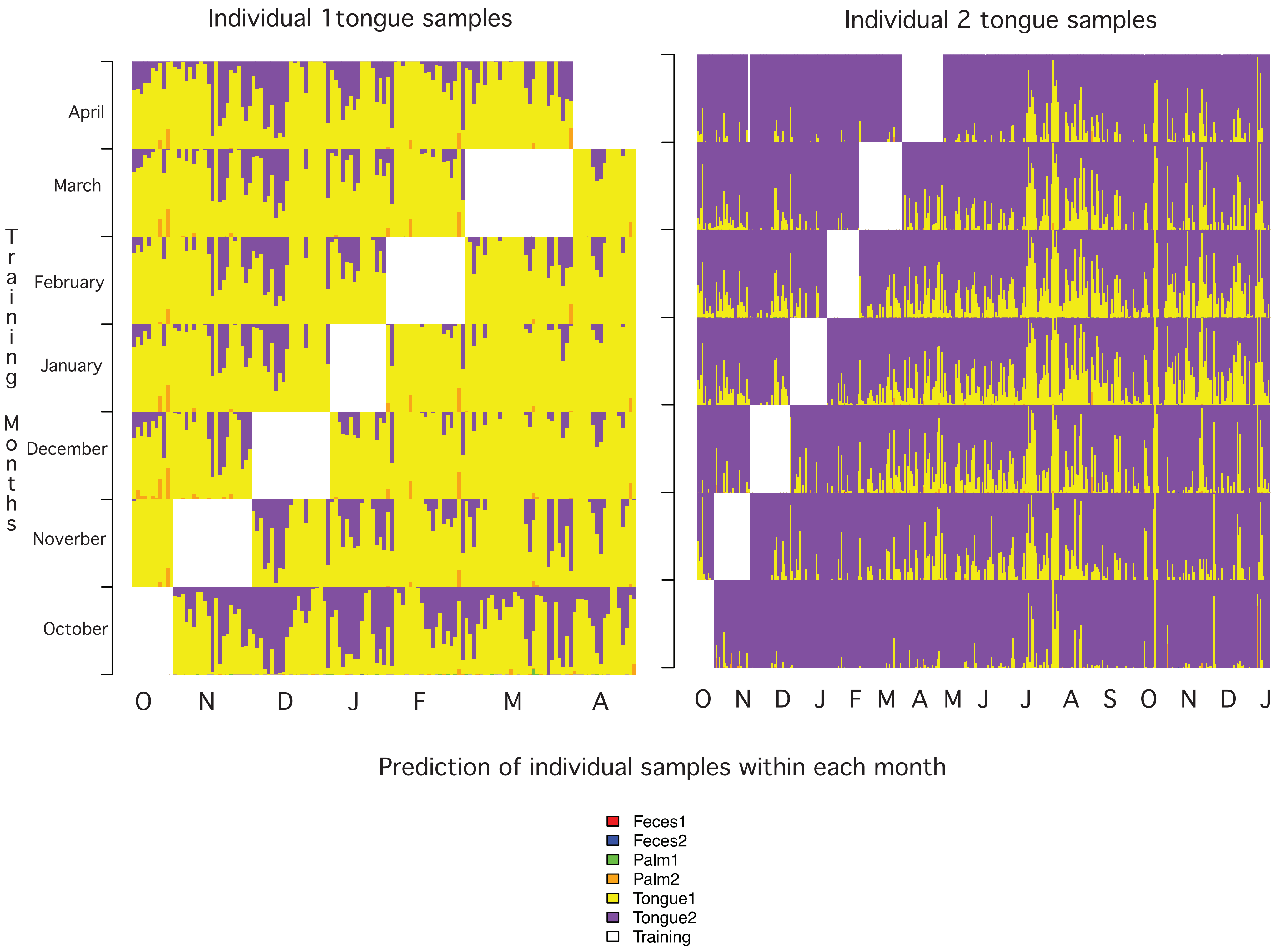

Individual 1tongue samples
Individual 2 tongue samples
Training Months
April
March
February
January
December
Noverber
October
O N D J F M A
O N D J F M A M J J A S O N D J
Prediction of individual samples within each month
Feces1
Feces2
Palm1
Palm2
Tongue1
Tongue2
Training
