# Supplementary material for: BioMiCo: a supervised Bayesian model for inference of microbial community structure
Source: Microbiome. 2015 Mar 10;3:8. doi: 10.1186/s40168-015-0073-x (PMC4359585; doi:10.1186/s40168-015-0073-x)
Supplement: Additional file 4: Figure S2. — This figure identifies the outlier samples that correspond to labeling errors. Fecal samples are shown in panel (A), palm samples are shown in panel (B), and tongue samples are shown in panel (C). The plots show the prediction of both human host identity and body site according to maximum posterior probability, with the outliers indicated by arrows and labeled with sample IDs. The original analysis by Caporaso et al. [1] excluded these outliers. Samples were from the gut, palm, and tongue of two individuals collected over 7 and 16 months (individuals 1 and 2, respectively) [1]. The white blocks within the plots are the months that were used to train the model. Every row corresponds to the results obtained from a different training month. The height for each row corresponds to the posterior probability scale of 0 to 1. Results for the gut samples from the same two individuals are provided in Figure 2. [file 40168_2015_73_MOESM4_ESM.pdf]

Figure S2 A

Mislabeled samples indicated with an arrow.
These samples were originally labeled as fecal samples and later determined to be mislabeled.

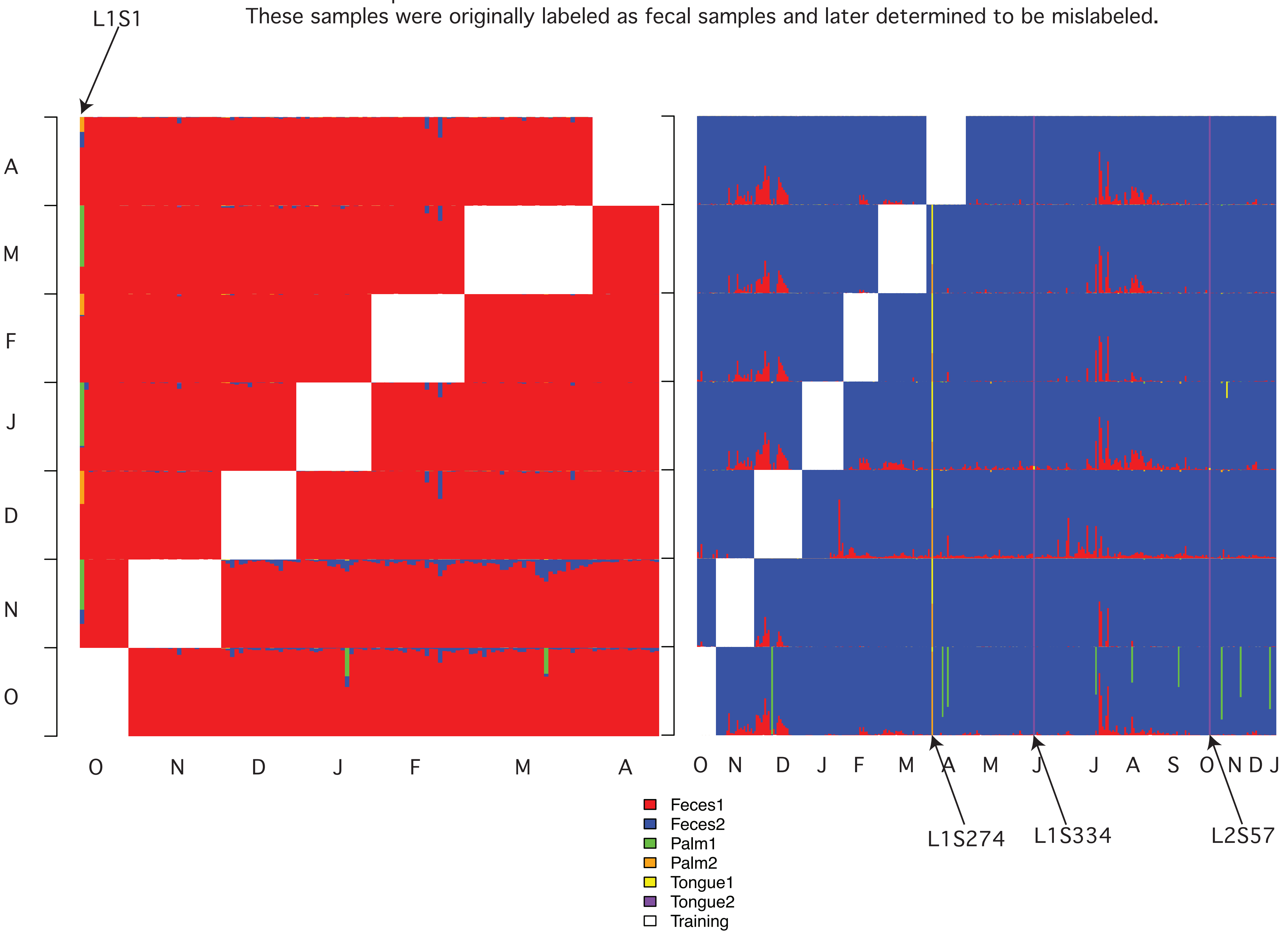

Figure S2 B

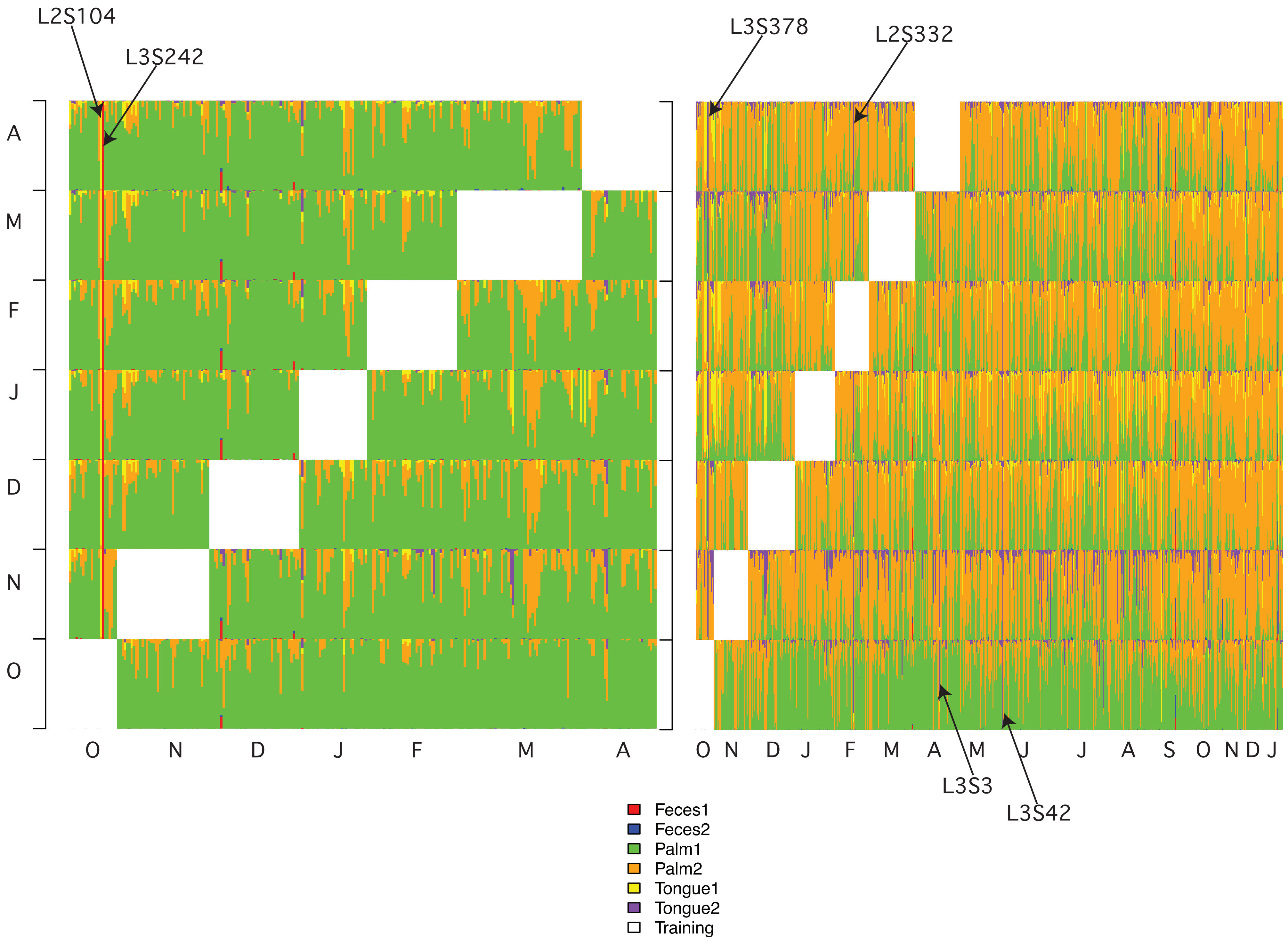

L2S104
L3S242
L3S378
L2S332
A
M
F
J
D
N
O
O
N
D
J
F
M
A
O
N
D
J
F
M
A
M
J
J
A
S
O
N
D
J
L3S3
L3S42
Feces1
Feces2
Palm1
Palm2
Tongue1
Tongue2
Training

Figure S2 C

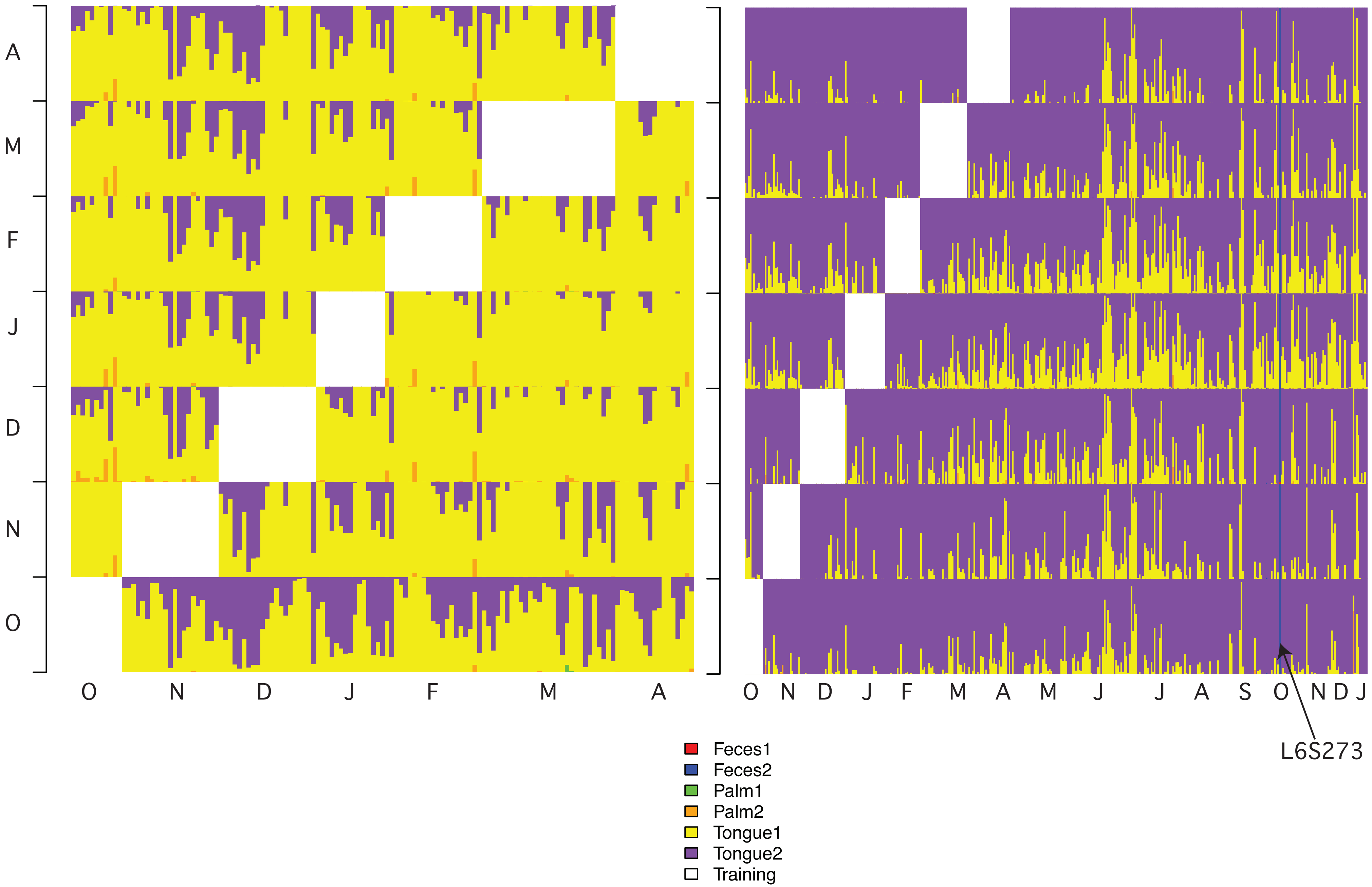

A
M
F
J
D
N
O
O
N
D
J
F
M
A
O N D J F M A M J J A S O N D J
L6S273
Feces1
Feces2
Palm1
Palm2
Tongue1
Tongue2
Training
